# Immunoproteasomes control activation of innate immune signaling and microglial function

**DOI:** 10.3389/fimmu.2022.982786

**Published:** 2022-10-06

**Authors:** Gonca Çetin, Maja Studencka-Turski, Simone Venz, Eileen Schormann, Heike Junker, Elke Hammer, Uwe Völker, Frédéric Ebstein, Elke Krüger

**Affiliations:** ^1^ Institute of Medical Biochemistry and Molecular Biology, University Medicine Greifswald, Greifswald, Germany; ^2^ Institute of Biochemistry, Charité – University Medicine Berlin, Berlin, Germany; ^3^ Interfaculty Institute of Genetics and Functional Genomics, University Medicine Greifswald, Greifswald, Germany

**Keywords:** microglia, CNS, proteostasis, neuroinflammation, UPS, immunoproteasome, proteomics

## Abstract

Microglia are the resident immune cells of the central nervous system (CNS) and play a major role in the regulation of brain homeostasis. To maintain their cellular protein homeostasis, microglia express standard proteasomes and immunoproteasomes (IP), a proteasome isoform that preserves protein homeostasis also in non-immune cells under challenging conditions. The impact of IP on microglia function in innate immunity of the CNS is however not well described. Here, we establish that IP impairment leads to proteotoxic stress and triggers the unfolded and integrated stress responses in mouse and human microglia models. Using proteomic analysis, we demonstrate that IP deficiency in microglia results in profound alterations of the ubiquitin-modified proteome among which proteins involved in the regulation of stress and immune responses. In line with this, molecular analysis revealed chronic activation of NF-κB signaling in IP-deficient microglia without further stimulus. In addition, we show that IP impairment alters microglial function based on markers for phagocytosis and motility. At the molecular level IP impairment activates interferon signaling promoted by the activation of the cytosolic stress response protein kinase R. The presented data highlight the importance of IP function for the proteostatic potential as well as for precision proteolysis to control stress and immune signaling in microglia function.

## Introduction

Thanks to their capacity of sensing changes in their environment such as pathogens and/or small protein flux perturbations, microglia largely contribute to the surveillance of the central nervous system (CNS) (1–3). As microglia are in fact macrophages of the brain, they are able to phagocytose protein aggregates and pathogens to clear the environment and protect the brain from possible danger (2–4). Therefore, microglial function heavily relies on protein homeostasis and mediation of innate immune signaling. Protein homeostasis (also referred to as proteostasis) secures a delicate balance between protein synthesis, quality control and degradation (5, 6). This is in particular important in the CNS, in which persistent disturbances of this process typically cause neurodegeneration. Unbalanced proteostasis reflected by intra- and extracellular protein deposits is frequently accompanied by oxidative stress, neuroinflammation and progressive degeneration of specific brain regions represent hallmarks of neurodegenerative disorders (5, 7, 8). Efficient protein breakdown systems and phagocytotic potential of microglia protect the brain against the accumulation of these defective proteins.

One major intracellular protein degradation system is the ubiquitin-proteasome system (UPS) (9). The principal route of the UPS comprises ubiquitin modification of target proteins by the thioester cascade involving E1, E2, and E3 enzymes and subsequent proteolytic degradation of the ubiquitylated targets by the proteasome (10). Proteasomes are multi-subunit enzyme complexes composed of two sub-complexes, the 19S regulatory particle and the 20S core particle (9–11). The 19S regulatory particle recognizes ubiquitylated proteins prior to ubiquitin removal, substrate unfolding and translocation into the 20S core proteasome, where it is degraded by the catalytic subunits of the 20S proteasome. Three of the β subunits in each β ring; β1, β2 and β5 harbor the three catalytic activities; caspase-like, trypsin-like and chymotrypsin-like activities, respectively (12). Upon infection or inflammation, catalytic subunits of the standard proteasome (SP); β1, β2 and β5 are replaced by the inducible subunits; β1i/LMP2, β2i/MECL-1, and β5i/LMP7, respectively to form an alternative isoform with the enhanced proteolytic capacity, the immunoproteasome (IP) (12, 13). As an adaptation mechanism to stress stimuli, cells can adapt the ratio between SP and IP expression levels.

As a response to proteotoxic stress within the endoplasmic reticulum (ER) lumen, cells trigger compensatory mechanisms such as unfolded protein response (UPR) (14). The signaling is induced by three ER receptors inositol-requiring protein 1α (IRE-1α), activating transcription factor 6 (ATF6), and protein kinase RNA-like ER kinase (PERK). Activation of UPR results in the splicing of the Xbp1 transcription factor mRNA by IRE-1α, cleavage of ATF6 by the Golgi apparatus proteases, and phosphorylation of eukaryotic translation initiation factor 2 alpha (eIF2α) by PERK to upregulate stress elements and to restore the proteostasis network (15–17). The integrated stress response (ISR) is a cytosolic adaptation mechanism to proteotoxic stress, which intersects with the PERK branch of UPR to shut-down protein synthesis (18, 19). All four kinases of ISR, general control non-derepressible 2 (GCN2), heme-regulated eIF2α kinase (HRI), double-stranded RNA-dependent protein kinase (PKR) and PERK, trigger a global translational arrest by the phosphorylation of eIF2α (15, 16).

In immune cells, the UPS preserves key contributions of innate and adaptive immunity including signal transduction to transcription factors such as nuclear factor κB (NF-κB), the control of interferon (IFN) signaling upon viral infection and MHC class I antigen processing (17). It has been previously reported that patients with impaired proteasomal function (SP and/or IP) exhibit systemic inflammation including neuroinflammation (20–23). To address the molecular link between proteasome impairment and neuroinflammation, we focused on microglia as a model, since they are in the first line of immune defense in CNS and constitutively express IP as well as SP. Yet, the impact of proteasome activity on the microglial cellular function and innate immunity of CNS is not well understood. We have recently shown that pharmacological inhibition of pan-proteasome activity (SP and IP) in microglia results in the induction of type I IFN response which depends on the IRE-1α arm of the UPR (24), however, the contribution of the IP remained unclear in this context. To this end, we used *Psmb8* (gene encoding β5i/LMP7 catalytic subunit of IP) knockout mice as an IP deficiency model and human microglia cell line C20 treated with β5i/LMP7 inhibitor as an alternative model for human microglia cells. All together, we indicate that IP impairment alters the microglial function and promotes sterile inflammation in the brain.

## Materials and methods

### Primary microglia isolation from C57BL/6 mice and cell culture

Primary microglia were isolated from wild-type and β5i/LMP7 knockout C57BL/6 mice (25) regardless of gender. Equal numbers of female and male mice were used in each experiment. Mice were group-housed under pathogen-free conditions on a 12h light/dark cycle and provided with food and water *ad libitum*. Animals were sacrificed by cervical dislocation. After tissue dissection, brains were carefully collected in Hanks’ balanced salt solution (With phenol red, without calcium and magnesium (Sigma)) and left on ice. Cluster of differentiation 11b positive (CD11b+) cells isolation from brain tissue was performed using the Neural Tissue Dissociation Kit (P) (Miltenyi Biotec) and the magnetic cell sorting technique using CD11b-labeled magnetic microbeads (Miltenyi Biotec) according to manufacturer’s instructions. Isolated CD11b+ cells were cultured in 3.5cm dishes pre-coated with 50μg/mL poly-L-lysine solution (Millipore) and with Dulbecco’s modified Eagle’s medium (PAN Biotech) supplemented with 10% FBS (PAN Biotech), 100U/mL penicillin, 100μg/mL streptomycin (PAN Biotech), and glucose 40% (B. Braun Mini-Plasco R connect) at the final concentration of 4,500 mg/L. Cells were cultured at 37°C, 95% humidity, and 5% CO_2_.

The immortalized human microglia cell line C20 (26) was cultured with Dulbecco’s Modified Eagle’s Medium/Nutrient Mixture F-12 Ham (PAN Biotech) supplemented with 10% FBS (PAN Biotech), 100 U/mL penicillin, 100μg/mL streptomycin (PAN Biotech) at 37°C, 95% humidity, and 5% CO_2_.

### Microglia treatments

C20 cells were subjected to treatments with either IP specific inhibitor or ISR pathway inhibitors. β5i/LMP7 activity was inhibited with different doses of ONX-0914 (APExBIO) for 24h or with a dose of 200nM of ONX-0914 for 4h, 12h or 24h. The treatment with the diluent only, dimethyl sulfoxide (DMSO) (0.01% v/v) was served as a control. PERK or PKR activities were inhibited by the treatment for 2h with different doses of GSK2606414 (MedChemExpress) or C16 inhibitor (Sigma Aldrich), respectively. 1µM of C16 was used for further experiments. The diluent only treatment DMSO (0.1% v/v) was used as a control. Cells were harvested, snap-frozen and kept at -80 °C for further analysis.

Primary microglia were either treated with 50nM water-soluble pan-proteasome inhibitor bortezomib (BTZ) (Velcade, Takeda Oncology) for 8h or stimulated with water-soluble 1µg/mL toll-like receptor 4 ligand lipopolysaccharide (LPS) (*In vivo*Gen) for 8h. Cells were harvested, snap-frozen and kept at -80 °C for further analysis.

### Cell viability

Cytotoxic effects of ONX-0914 and C16 on C20 cells were determined by flow cytometry. After treatments, cells were harvested with trypsin and resuspended in PBS (attached cells and non-attached cells in the medium were combined) and, stained with 1:100 dilution of 4′,6-diamidino-2-phenylindole (DAPI) (Miltenyi Biotec). The live/dead cell determination was performed using flow cytometry (MACS Quant Analyzer 10, Miltenyi Biotec). Cells were gated by forward scatter area and side scatter area and, singlets were selected. DAPI positive signal was detected in V1-VioBlue channel. DAPI negative cells were counted as viable cells. The results were analyzed using MACSQuantify (Miltenyi Biotec).

### Western blotting

Frozen cell pellets were resuspended in RIPA buffer [50mM Tris pH 7.5, 150mM NaCl, 2mM EDTA, 1% (v/v) NP-40, 0.1% sodium dodecyl sulfate, 1mM Na_3_VO_4_, 10mM NaF, 2mM Na_4_P_2_O_7_, 10μM MG132, 10mM N-ethylmaleimide (NEM), and EDTA free protease inhibitor cocktail (Roche)], vortexed and incubated on ice for 30 min. Samples were centrifuged at 4°C for 10 min at 11,000 rpm and supernatants were collected and stored at -20°C for further analysis. Protein concentration was determined using the Bicinchoninic Acid (BCA) Assay Kit (Thermo Scientific).

Protein fractionation was performed to detect nuclear translocation of protein in interest. Primary microglia cell pellets were resuspended in TSDG buffer [10mM Tris pH 7.5, 10mM NaCl, 25mM KCl, 1mM MgCl_2_, 0.1mM ethylenediaminetetraacetic acid (EDTA), 2mM dithiothreitol, 10% (v/v) glycerin] and underwent four cycles of freezing and thawing using liquid nitrogen and ambient water. Samples were centrifuged at 4°C for 20 min at 15,000 × g; supernatants were collected and labelled as cytosolic fractions. Remaining cell pellets were resuspended in urea buffer (8M urea, 2M thiourea, 4% CHAPS, 40mM Tris base and freshly added 50mM DTT), vortexed, incubated on ice for 15 min and, samples labelled as nuclear fractions. Protein concentration was determined using the Bradford Assay Kit (Thermo Scientific).

Depending on the protein of interest, different amounts (10, 20 or 40 μg) of the protein samples were loaded onto Laemmli sodium dodecyl sulfate (SDS) polyacrylamide gels and separated by electrophoresis. Proteins were transferred by wet electroblotting to the PVDF membranes (Millipore). The membranes were blocked with 1× Roti R-Block (Carl Roth) and incubated at 4°C overnight with the following primary antibodies provided in **Supplementary Table 1**. After incubation with primary antibodies, membranes were washed with 1× tris-buffered saline containing 0.1% Tween-20 (TBST) and incubated with peroxidase-conjugated anti-rabbit IgG or anti-mouse IgG secondary antibodies (**Supplementary Table 1**) for 1 hour at room temperature. After removal of excessive antibodies by washing with TBST, membranes were incubated with peroxidase substrate solution ECL Clarity (Bio-Rad). The signal intensities were determined using X-ray films or the FUSION FX imaging system (Vilber Lourmat). Western blot data were quantified using ImageJ software and all data normalized to respective gel loading controls. All Western blotting data are derived from at least three biological replicates and represent one experiment with similar results.

### RNA isolation, reverse transcription and quantitative real-time polymerase chain reaction

RNA was isolated using the innuPREP RNA Mini Kit (Analytik Jena) according to the manufacturer’s protocol. Complementary DNA was synthesized using the M-MLV Reverse Transcriptase (Promega) according to the manufacturer’s instructions. Quantitative real-time PCR (qRT-pCR) was performed either with the TB Green Premix Ex Taq polymerase (Takara Clontech) or the TaqMan probe Ex Taq polymerase (Takara Clontech) and using a CFX96 TouchTM Real-Time PCR Detection System (Bio-Rad). Relative quantification was normalized by reference genes. Primers and TaqMan probes used for the qRT-PCR are provided in Supplementary Material (**Supplementary Tables 2** and **3**, respectively).

### Enzyme-linked immunosorbent assay

After isolation of primary microglia from wild-type and β5i/LMP7 knockout C57BL/6 mice, cells were cultivated as described above and, then supernatants were collected. The pro-inflammatory cytokine interleukin-6 (IL-6) was quantified by using the Mouse IL-6 High-Sensitivity ELISA kit (Invitrogen). Data were analyzed according to manufacturer’s instructions.

### Proteasome active sites determination by flow cytometry

Proteasome activity of wild-type and β5i/LMP7 knockout primary microglia or human C20 microglia treated with ONX-0914 was determined after incubation with a 500nM pan-reactive fluorescent proteasome activity-based probe (ABP) Me4BodiPyFL-Ahx3Leu3VS (UbiQ-018) in culture media for 2 hours at 37°C, 95% humidity, and 5% CO_2_. After treatment, cells were collected and resuspended in phosphate buffer saline (PBS) (PAN Biotech). Primary microglia were additionally stained with 1:100 dilution of 4′,6-diamidino-2-phenylindole (DAPI) (Miltenyi Biotec) for dead cell exclusion. Proteasome active sites were determined by flow cytometry (MACS Quant Analyzer 10, Miltenyi Biotec). Cells were gated by forward scatter area and side scatter area and, singlets were selected. ABP BodiPy FL signal was detected in B1-FITC channel. The results were analyzed using MACSQuantify (Miltenyi Biotec).

### Microglial markers determination by flow cytometry

To quantify microglia population in the brain, cell suspensions obtained after brain dissociation were stained with CD11b FACS antibody (130-113-803, Miltenyi Biotec) or isotype control human IgG1 (130-120-709, Miltenyi Biotec) according to the manufacturer’s instructions. After staining, cells were washed with PBS and collected and resuspended with 1mL of PBS. Cells were gated by forward scatter area and side scatter area and, singlets were selected. CD11b+ cells were detected in R1-APC channel. The results were analyzed using MACSQuantify (Miltenyi Biotec).

After magnetic cell sorting described above (Primary Microglia Isolation), CD11b+ cells were washed with PBS and, stained with CD11b FACS antibody (130-113-803, Miltenyi Biotec) to confirm purity of MACS isolation. After staining, cells were washed with PBS and collected and resuspended with 500µL of PBS. Cells were gated by forward scatter area and side scatter area and, singlets were selected. CD11b+ cells were detected in R1-APC channel. MACS isolated CD11b cells were then stained with CD68 antibody (12-0681-80, eBioScience) or isotype control rat IgG2a κ. Cells were gated by forward scatter area and side scatter area and, singlets were selected. CD68+ cells were detected in B2-PE channel. Flow cytometry was performed using MACS Quant Analyzer 10, Miltenyi Biotec. The results were analyzed using MACSQuantify (Miltenyi Biotec).

### Microglial phagocytosis determination by flow cytometry

Oligomeric amyloid-beta (Aβ) was prepared according to Klein et al. In-house generated Aβ peptide 1-42 (Aβ_42_) was dissolved in ice-cold 1,1,1,3,3,3-Hexafluoro-2-propanol (HFIP) (Fluka Analytical) to a ratio of 2.5mg peptide/1 mL HFIP. The peptide/HFIP mix was then incubated for 1h at room temperature followed by 1h incubation on ice for monomerization. The monomerized Aβ_42_ was further aliquoted in low-bind Eppendorf tubes 125μg and HFIP was evaporated overnight. Residual HFIP was eliminated using a SpeedVac for 10min. The resulting peptide film was then stored at -80°C. To generate oligomeric Aβ, the peptide film was solved in 5.5μL Dimethyl Sulfoxid (DMSO) (Applichem) thoroughly and further diluted in phenol-red free Dulbecco’s Modified Eagle Medium Nutrient Mixture F-12 (DMEM/F12, (+) L-Glutamin) (Gibco) to reach an Aβ concentration of approximately 100μM. For the analysis in confocal microscopy, 6μg tetramethylrhodamine (TAMRA)-labeled Aβ42 (AnaSpec) dissolved in DMSO was added to the DMSO-solved Aβ prior to the dilution in DMEM/F12 to reach a fraction of 5% TAMRA-Aβ. After 16h of oligomer formation in the cold room at 4°C the peptide solution was centrifuged at 14 000 g for 15min at 4°C to remove fibrillary Aβ. The effective peptide concentration in the supernatant was determined using the bicinchoninic acid (BCA) protein assay Kit (Thermo Fisher) and 2μM Aβ oligomers were used for treatments.

Isolated wild-type and β5i/LMP7 knockout primary microglia were incubated with 2µM of TAMRA-labeled Aβ oligomers for 1 hour and then, cells were collected and stained with CD45 antibody (130-110-803, Miltenyi Biotec) or isotype control human IgG1 (130-113-456, Miltenyi Biotec) according to the manufacturer’s instructions. Cells were washed with PBS and collected and resuspended with 500µL of PBS. Cells were gated by forward scatter area and side scatter area and, singlets were selected. Aβ+ and CD45+ cell populations were determined B2-PE and V2-VioGreen channels, respectively, by flow cytometry (MACS Quant Analyzer 10, Miltenyi Biotec). The results were analyzed using MACSQuantify (Miltenyi Biotec).

### Fluorescence microscopy

Isolated wild-type and β5i/LMP7 knockout primary microglia were cultured on sterile 24 mm x 24 mm coverslips pre-coated with 50 μg/mL poly-L-lysine solution (Millipore) in a 6-well plate at 37°C, 95% humidity, and 5% CO_2_. After 1 week culture, cells were fixed with 4% paraformaldehyde in pH=7.4 phosphate buffer for 15 min at room temperature. Cells were subsequently washed three times with PBS and, permeabilized and blocked for 30 min with immunofluorescence (IF) buffer containing 0.2% BSA, 0.05% saponin, 0.1% NaN3 in PBS. Cells were incubated overnight at 4°C with NF-κB (Santa Cruz, sc-372) antibody. After primary antibody incubation, cells were washed three times with IF buffer and incubated with anti-rabbit Alexa Fluor 488 (Invitrogen A-11034 or 11001) and rhodamine phalloidin (Life Technologies) for 1 hour at room temperature. After removal of excessive antibody by washing three times with IF buffer and once with PBS, cells were stained with DAPI. Coverslips were transferred onto microscopy slides covered with Fluorescent Mounting Medium (Dako). Images were collected using Leica TCS SP5 System (Leica Microsystems CMS GmbH) equipped with a Suite Advanced Fluorescence application (LAS AF 2.7.3.9723) at ×63 magnification. Co-localization analysis of NF-κB and nuclear staining was evaluated by Pearson’s Coefficient correlations using LAS AF 2.7.3.9723 software (Leica Microsystems CMS GmbH).

Organotypic brain slice cultures were prepared from postnatal 3–6 day-old mice pups. Brains were rapidly dissected and separated in hemispheres. The cut side was glued to the metal block of the vibratome (Leica VT1200S), which was then placed in cold Hank’s balanced salt solution (HBSS, Gibco) supplemented with 1% penicillin/streptomycin (Pan Biotech), 0.6% glucose (Fluka), and 20 mM HEPES (Sigma-Aldrich), purged with Carbogen for dissection in sagittal slices of 300μm. Cerebellum was removed using a razor blade. Slices were collected and placed onto membrane inserts (Millicell, 0.45μm; Merck Millipore) in six-well plates containing 1mL of minimum essential medium Eagle (Gibco) supplemented with 25% horse serum (Gibco), 20.7% HBSS, 1% P/S, 0.6% glucose, and 2% B27 (Gibco). Two slices per insert were cultivated at 35°C and 5% CO_2_ for 8 days before staining. Organotypic brain slice cultures were incubated with 500nM pan-reactive fluorescent proteasome activity-based probe (ABP) Me4BodiPyFL-Ahx3Leu3VS (UbiQ-018) at 35°C for 1 hour. In a 24 well plate, slices were subsequently fixed in cold 4% paraformaldehyde shaking for 20 min at room temperature, washed in 1x PBS-1% Triton-X for 20min. PBS-T was replaced by MilliQ H_2_O and immediately replaced by autofluorescence reduction solution (100mM CuSO_4_/50mM CH_3_COONH_4_). After 1 h incubation, the solution was replaced by MilliQ H_2_O and then by 1x PBS. Finally, slices were mounted in Fluoromount (aqueous, Sigma Aldrich) using the bridging technique. Thereby, two cover slips were glued onto the microscope slide, the slice in mounting medium placed between and covered by the final cover slip. After resting for 1 h, the edges were sealed using transparent gel nail polish. Brain slices were further imaged using the Nikon A1r+ confocal microscope setup (AMBIO facility, Charité) using a 20x objective. Z-stacks were obtained with 0.1μm step size. Images were further processed in Image J software.

Microtubule-associated protein 2 (MAP2) staining for neurons and allograft inflammatory factor 1 (IBA-1) staining for microglia were performed in a 24 well plate. Organotypic brain slice cultures were subsequently fixed in cold 4% paraformaldehyde shaking for 20 min at RT, washed in 1x PBS/1% Triton-X for 20 min and incubated in blocking solution (10% normal goat serum (NGS), 0.3% Triton-X in 1x PBS [PBS-T]) shaking for 2 hours at RT Slices were then incubated in anti-MAP2 (188004, SynSys) and anti-IBA-1 (019-19741, Wako) antibodies diluted 1:500 in PBS-T/5% NGS for 48 hours shaking in the cold room. Further, slices were washed twice for 30 min at RT and followed by overnight shaking in the cold room in PBS-T. The next day, slices were washed again twice for 30 min at RT in PBS-T followed by incubation in secondary antibody diluted 1:500 in PBS-T/5% NGS for 24 hours shaking in the cold room. The following day, slices were washed 6x for 30 min in PBS-T at RT. PBS-T was replaced by MilliQ H_2_O and immediately replaced by autofluorescence reduction solution (100 mM CuSO_4_/50mM CH_3_COONH_4_). After 1 hour of incubation the solution was replaced by MilliQ H_2_O and then by 1x PBS. Finally, slices were mounted in Fluoromount (aqueous, Sigma Aldrich) using the bridging technique. Thereby, two cover slips were glued onto the microscope slide, the slice in mounting medium placed between and covered by the final cover slip. After resting for an hour, the edges were sealed using transparent gel nail polish. Immunostained slices were further imaged using the Nikon A1r+ confocal microscope setup (AMBIO facility, Charité). The hippocampal region of interest was examined using a 60x objective. Z-stacks were obtained with 0.1 µm step size. Images were further processed in Image J. IBA-1^+^ microglia were counted using the “analyze particle” function (applied on SUM stacks). Thresholds were set equally for every image analyzed.

### Enrichment of ubiquitylated proteins for mass spectrometry

Wild-type and β5i/LMP7 knockout primary microglia cell pellets were resuspended in TSDG buffer (10mM Tris-HCl pH 7.5, 25mM KCl, 10mM NaCl, 1mM MgCl_2_, 0,1mM EDTA, 10% (v/v) glycerol, 1mM DTT, 10mM NEM, 10μM MG132) and lysed by four cycles of freezing and thawing using liquid nitrogen and ambient water. Samples were centrifuged at 4°C for 20 min at 15,000x-g, supernatants were collected for further analysis. Protein concentration was determined using the Bradford method. Endogenous ubiquitylated proteins were isolated using the Ubi-Qapture-Q kit (Enzo Life Sciences) as described by the manufacturer. TSDG protein samples were incubated with the Ubi-Quapture-Q matrix at 4°C for 4 hours under horizontal shaking. Captured samples were washed twice with PBS, eluted with the mixture of SDS-PAGE sample buffer (20%) and PBS (80%), elution was verified by Western blotting.

### Liquid chromatography-tandem mass spectrometry LC-MS/MS and protein identification

Ubiquitylated proteins were identified by liquid chromatography-tandem mass spectrometry (LC-MS/MS). Protein samples were prepared for the LC-MS/MS measurement using single pot solid-phase enhanced sample preparation (SP3) technology. Proteins were reduced with 25 mM DTT in 20 mM Tris buffer at 37°C for 30 min, alkylated with 100 mM iodoacetamide in 20 mM Tris buffer at 37°C for 15 min and, quenched using 25 mM DTT. Then, proteins were capped using magnetic Sera-Mag Speedbeads (1:1, hydrophobic: hydrophilic) (Thermo Scientific) and precipitated with acetonitrile (ACN) at room temperature for 18 min and constant shaking at 1400 rpm. After washing the beads with 70% (v/v) ethanol and ACN, beads were air-dried at room temperature, reconstituted in 20mM ammonium bicarbonate. Beads-bound proteins were digested with 20ng/µL trypsin at 37°C for 16 hours (enzyme to protein ratio 1:25). Digestion was ended up with ACN addition and incubation at room temperature for 18 min and constant shaking at 1400 rpm. Peptides were eluted with 2% DMSO and 2x concentrated buffer A (4% ACN and 0.2% acetic acid in H_2_O). Description of experiment settings for LC-MS/MS analysis and presentation of protein identification are provided in supplementary data (**Supplementary Table 4**).

Protein identification and quantification were performed using Proteome Discoverer 2.4 (Thermo Scientific). Functional enrichment analysis of identified proteins was performed using g:Profiler (https://biit.cs.ut.ee/gprofiler/gost) and GeneCodis 4.0 (https://genecodis.genyo.es/). Heatmaps were created using Morpheus software (https://software.broadinstitute.org/morpheus).

### Statistical analysis

Results for primary microglia are given the mean of at least three experiments ± standard error of the mean (SEM) in which four or five mice from both genotypes were used and isolated microglia were pooled in each experiment. The data with C20 cells are the mean of at least three independent biological replicates ± SEM. The statistical significances of all experiments were evaluated with paired t-tests by using GraphPad Prism 8.0.2.

## Results

### β5i/LMP7 impairment attenuates proteasomal activity and subsequently leads to proteotoxic stress in microglia

**Figure 1:**
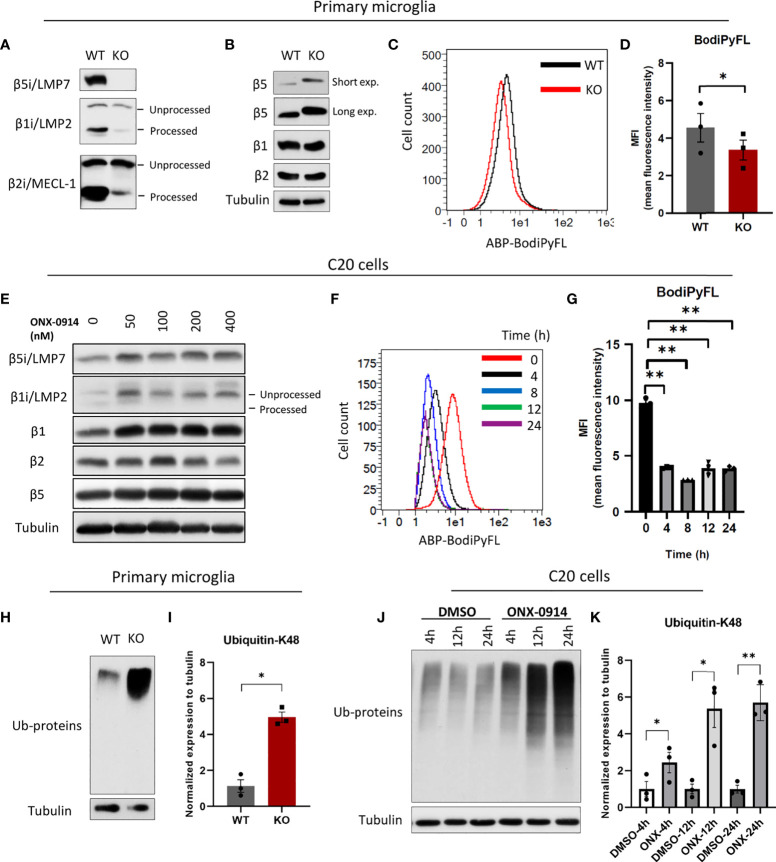
Immunoproteasome impairment disturbs protein homeostasis in microglia. **(A)** Western blot analysis of immunoproteasome catalytic subunits in WT and β5i/LMP7 KO primary microglia. **(B)** Western blot analysis of standard proteasome catalytic subunits in WT and β5i/LMP7 KO primary microglia. **(C)** Primary murine microglia were incubated with 500nM of pan-reactive proteasome activity-based probe Me4BodiPyFL-Ahx3Leu3VS (ABP) for 2 hours to stain proteasome active sites, histograms showing FITC-ABP signals in WT (black) and KO (red) microglia. **(D)** Bar graph showing the comparison of proteasome active sites in WT and β5i/LMP7 KO primary microglia as indicated by MFI (mean fluorescence intensity) of FITC-ABP Me4BodiPyFL-Ahx3Leu3VS. **(E)** Western blot analysis of proteasome catalytic subunits in human microglia C20 cells treated with different doses of ONX-0914 for 24h. **(F)** Histograms showing FITC-ABP signals in C20 cells untreated (red) or treated with 200nM ONX-0914 for 4h (black), 8h (blue), 12h (green) and 24h (purple). **(G)** Bar graph showing the comparison of proteasome active sites in C20 cells untreated or treated with 200nM ONX-0914 for indicated time (MFI (mean fluorescence intensity) of FITC-ABP Me4BodiPyFL-Ahx3Leu3VS). **(H)** Western blot analysis of poly-ubiquitylated proteins in WT and β5i/LMP7 KO primary microglia. **(I)** Quantification of Western blot data from figure 1H. **(J)** Western blot analysis of poly-ubiquitylated proteins in C20 cells treated with 200nM ONX-0914 or DMSO for indicated time. **(K)** Quantification of Western blot data from figure 1J. All data are given (n=3) by mean ± SEM; *: P < 0.05, **: P < 0.01.

Given that β5i/LMP7 is essential for removal of the pro-peptides from other catalytic subunits during IP assembly (12), we here show that β5i/LMP7-deficient microglia were mostly devoid of IP, as evidenced by the inability of these cells to process the β1i/LMP2 and β2i/MECL-1 inducible subunits (**Figure 1A** and **Supplementary Figure 1A**). We also observed increased protein expression of β5, the SP counterpart of β5i/LMP7, which is known to compensate for decreased chymotryptic-like activity as observed in other settings [**Figure 1B** and **Supplementary Figure 1A**; (27)]. In addition, IP-deficient microglia displayed moderately decreased proteasomal activity as indicated by reduced binding of pan-reactive proteasome activity-based probe (ABP) Me4BodiPyFL-Ahx3Leu3VS (**Figures 1C, D** and **Supplementary Figure 1B**). Additionally, we stained organotypic brain slice cultures from wild-type and IP-deficient mouse pubs with the same ABP and confirmed the impaired total proteasome activity near *in vivo* conditions (**Supplementary Figures 1C, D**). In line with this data, β5i/LMP7 inhibitor ONX-0914 treatment in C20 cells restricted the processing of β1i/LMP2 (**Figure 1E**; **Supplementary Figures 2A, B**) and decreased activity-based probe binding as determined by Western blotting and flow cytometry, respectively (**Figures 1F, G** and **Supplementary Figure 2C**). At the same time, cytotoxicity of ONX-0914 on C20 cells was examined and, non-toxic conditions of ONX-0914 were used for all experiments (**Supplementary Figure 2D**).

**Figure 2:**
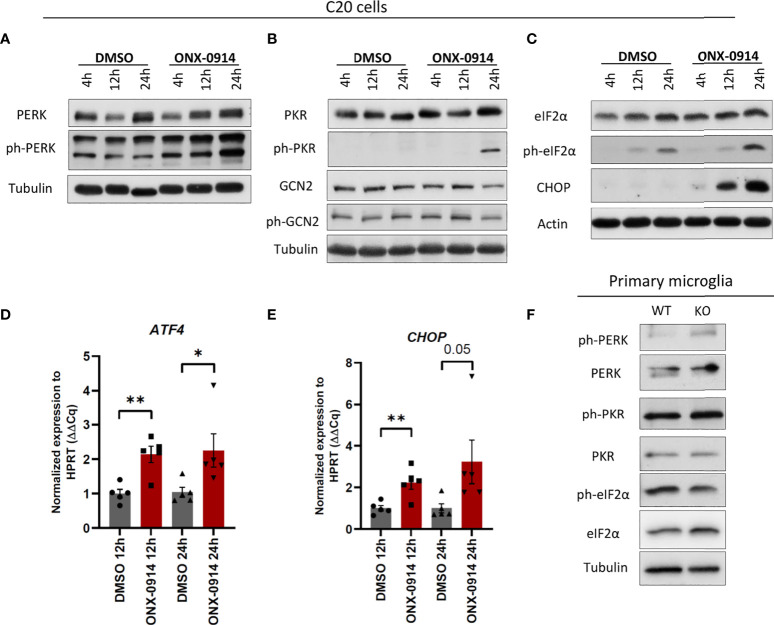
β5i/LMP7 inhibition activates UPR/ISR in microglia. **(A–C)** Western blot analysis of UPR and ISR proteins in C20 cells treated with 200nM of ONX-0914 for indicated time (n=3). **(D, E)** Quantitative reverse transcription PCR (RT-PCR) analysis of ATF4 **(D)** and CHOP **(E)** mRNA levels in C20 cells treated with 200nM of ONX-0914 for indicated time (n=5). **(F)** Western blot analysis of UPR and ISR proteins in WT and β5i/LMP7 KO primary microglia (n=3). Mean ± SEM; *: P < 0.05, **: P < 0.01.

We next investigated the impact of β5i/LMP7 deficiency on microglial proteostasis. IP dysfunction resulted in the generation of proteotoxic stress as indicated by accumulation of poly-ubiquitylated proteins in β5i/LMP7 knockout (KO) primary murine microglia (**Figures 1H, I**) as well as in human microglia C20 cells exposed to β5i/LMP7 specific inhibitor ONX-0914 (**Figures 1J, K** and **Supplementary Figures 3A, B**). In response to proteotoxic stress, cells typically activate compensation mechanisms such as UPR and ISR (14, 18). As shown in **Figures 2A–E** and **Supplementary Figure 4A**, inhibition of IP activity in microglia induced UPR and ISR activation to counteract proteotoxic stress. The treatment with ONX-0914 of C20 cells activated the PERK arm of UPR as indicated by the phosphorylation of PERK and its downstream target eIF2α (**Figures 2A, C** and **Supplementary Figures 3C, D** and **4A**). However, activation of the other branches of UPR was not observed (**Supplementary Figure 3E**). Inhibition of eIF2α by the phosphorylation mechanism results in a global translation arrest and this is also triggered by the other kinases of ISR including PKR and GCN2 (15, 16). In fact, we observed that the PKR branch of ISR was also activated in C20 cells following ONX-0914 treatment (**Figure 2B**) whereas no activation of GCN2 was observed under these conditions (**Figure 2B**). As expected, phosphorylation of eIF2α by PERK and PKR induced expression and activation of transcription factors including activating transcription factor 4 (ATF4) and its downstream target C/EBP homologous protein (CHOP) (14, 15, 18) in ONX-0914 treated microglia (**Figure 2C–E**). Interestingly, β5i/LMP7 KO induced PERK activation but did not affect PKR (**Figure 2F** and **Supplementary Figure 4B**). Despite activation of PERK in β5i/LMP7 KO microglia, eIF2α phosphorylation in primary murine microglia was permanently activated (**Figure 2F**). Altogether, these data indicate that our β5i/LMP7 impairment models behave differently with respect to their ability to engage compensatory mechanisms in response to proteotoxic stress.

### Diminished immunoproteasome activity induces accumulation of ubiquitylated proteins involved in immune responses

**Figure 3:**
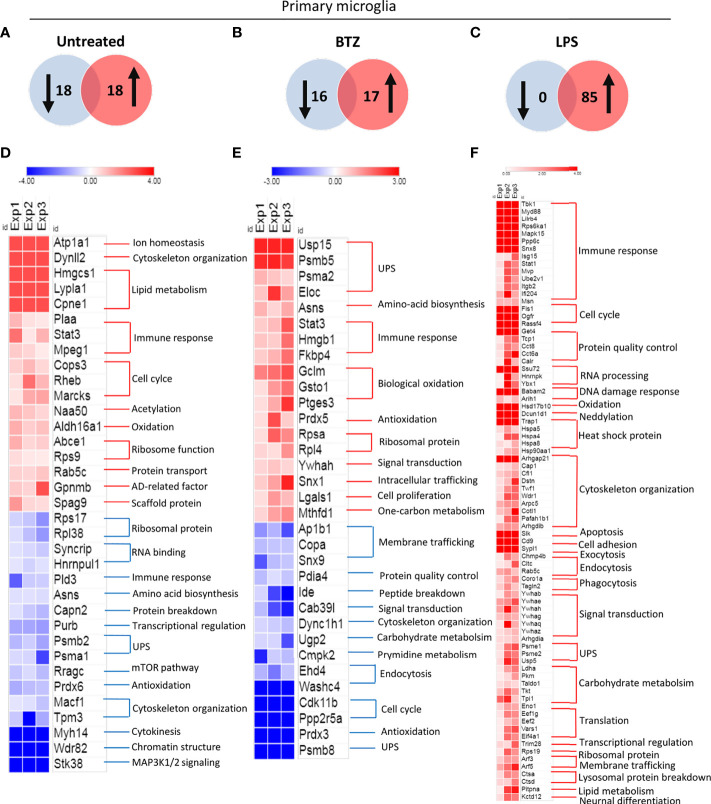
Immunoproteasome dysfunction alters the profile of ubiquitylated proteins in primary microglia identified by proteomics. Identified ubiquitylated proteins were compared in WT and β5i/LMP7 KO microglia by the fold changes of protein abundances. **(A–C)** Venn diagrams showing identified ubiquitylated protein numbers decreased (left, blue) and increased (right, red) in untreated **(A)**, 8h 50nM BTZ treated **(B)**, and 8h 1µg/mL LPS stimulated **(C)** β5i/LMP7 KO primary microglia in comparison to WT untreated, 8h 50nM BTZ treated, and 8h 1µg/mL LPS stimulated primary microglia, respectively. **(D–F)** Heatmaps showing proteins increased (red) and decreased (blue) in untreated **(D)**, 8h 50nM BTZ treated **(E)**, and 8h 1µg/mL LPS stimulated **(F)** β5i/LMP7 KO primary microglia in comparison to WT untreated, 8h 50nM BTZ treated, and 8h 1µg/mL LPS stimulated primary microglia, respectively. Heatmap data are given by log2 values of fold changes in protein abundances ((n=3) fold change cut off 1,3).

We next sought to determine whether microglia lacking β5i/LMP7 show alterations in protein ubiquitylation under basal and challenging conditions. To this end, we subjected WT and β5i/LMP7 KO primary microglia to treatment with bortezomib (BTZ, inhibits overall chymotrypsin-like activity) and toll-like receptor (TLR) 4 ligand lipopolysaccharide (LPS, mimics bacterial infection and stimulates inflammation). As shown in **Supplementary Figure 5**, exposure of the cells to either BTZ or LPS resulted in increased accumulation of ubiquitin-modified proteins in both cell types, albeit to a much stronger extent in β5i/LMP7 KO microglia. BTZ treatment and LPS stimulation increased the accumulation of ubiquitylated proteins in β5i/LMP7 KO microglia as well as WT microglia (**Supplementary Figure 5B**). However, the increase for all settings was pronounced in β5i/LMP7 KO microglia (**Supplementary Figure 5B**). In an attempt to identify the proteins preferentially degraded by the IP and to investigate the impact of the accumulation of these proteins on microglia function, we performed a mass spectrometry-based proteomic approach and functional pathway enrichment analysis. Due to the low stoichiometry of ubiquitylated proteins in the proteome, we used an enrichment technique (**Supplementary Figures 5A–D**) to identify ubiquitylated proteins. As summarised in **Supplementary Figure 6A**, to characterize the enriched proteins, we followed trypsin based in-liquid digestion approach, which prevents the material loss, prior to liquid chromatography-tandem mass spectrometry (LC-MS/MS) and proteomic analysis using Proteome Discoverer 2.4. As shown in **Supplementary Figure 6B**, the vast majority of identified proteins were common to all three biological replicates, thereby supporting the reliability of our experimental setup. While the number of identified proteins did not substantially vary between WT and β5i/LMP7 KO under basal conditions and following BTZ treatment (**Figures 3A, B** and **Supplementary Figures 6C, D**), it was substantially higher in β5i/LMP7 KO in response to LPS (**Figure 3C** and **Supplementary Figure 6E**), suggesting that these cells were more vulnerable to TLR signaling (28, 29) than their wild-type counterparts. Next, we performed a functional pathway analysis of altered ubiquitylated proteins in untreated, BTZ treated and LPS stimulated β5i/LMP7 KO microglia based on fold changes calculated by abundances of proteins in comparison to relative WT controls. β5i/LMP7 KO deficiency in primary microglia affected the ubiquitylation of proteins involved in multiple pathways (**Figures 3D-F**). Strikingly, immune response, cytoskeleton organisation, UPS, cell cycle and ribosomal proteins were the most affected pathways (top 5) by β5i/LMP7 deficiency in microglia (**Supplementary Figure 6F**). We further analyzed the ubiquitin-modified proteins accumulated in β5i/LMP7 KO microglia, which are most likely specific proteolytic targets of IP. The analysis revealed that the proteins preferentially degraded by IP were mostly involved in immune responses, cytoskeleton organisation, signal transduction, cell cycle and energy metabolism pathways (**Supplementary Figure 6G**). Specifically, ubiquitin-modified proteins which were enriched in β5i/LMP7 KO microglia following LPS stimulation include many proteins related to immune signaling (**Figure 3F**). Taken together, proteomics data showed that IP have an important role in the turnover of proteins involved in immune signaling in microglia and thus controling their activity.

### β5i/LMP7 deficiency results in accumulation of STAT3 and increased of NF-κB signaling in primary murine microglia

**Figure 4:**
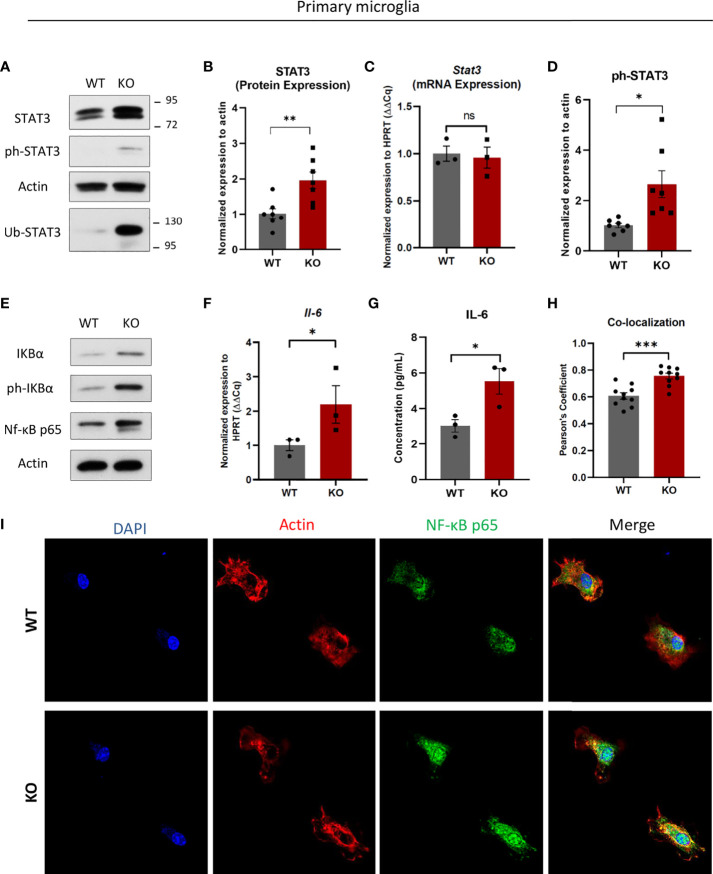
Immunoproteasome deficiency results in accumulation of signal transducer and activator of transcription 3 (STAT3) and increase of nuclear factor-kappa B (NF-κB) pathway proteins in primary murine microglia. **(A)** Western blot analysis of signal transducer and activator of transcription 3 (STAT3) in WT and β5i/LMP7 KO primary murine microglia. **(B)** Quantification analysis of STAT3 Western blot data from figure 4A (n=7). **(C)** Quantitative reverse transcription PCR (RT-PCR) analysis of *Stat3* mRNA level in WT and β5i/LMP7 KO microglia (n=3). **(D)** Quantification analysis of ph-STAT3 Western blot data from figure 4A (n=7). **(E)** Western blot analysis of nuclear factor-kappa B (NF-κB) pathway proteins inhibitor kappa B (IκB), phosphoylatated IκB and NF-κB p65 (n=4). **(F)** Quantitative reverse transcription PCR (RT-PCR) analysis of interleukin-6 *(Il-6)* mRNA level in WT and β5i/LMP7 KO microglia (n=3). **(G)** Quantitative analysis of secreted interleukin-6 (IL-6) protein level in WT and β5i/LMP7 KO microglia as indicated by ELISA analysis (n=3). **(H)** Co-localization analysis of NF-κB and nuclear staining shown by Pearson’s Coefficient correlations in WT and β5i/LMP7 KO microglia (n=10). **(I)** Confocal fluorescence microscopy analysis of NF-κB p65 (AlexaFluor 488, Green) in nucleus (DAPI, blue) and cytosol (Actin, red) in WT and β5i/LMP7 KO microglia. Mean ± SEM; *: P < 0.05, **: P < 0.01, ***: P < 0.001. ns, not significant.

One interesting and potential IP target, Signal Transducer and Activator of Transcription 3 (STAT3) was more abundant in the IP-deficient setting (**Figure 3**). STAT3 is a transcription factor inducing the expression of several cytokines and chemokines (30). On the other hand, STAT3 can also interact with another transcription factor Signal Transducer and Activator of Transcription 1 (STAT1) and thereby has a fine-tuning activity on type I IFN response (31). STAT3 protein levels were elevated in β5i/LMP7 KO microglia, whereas its mRNA expression remained unchanged (**Figures 4A–C**). Importantly, we also observed an increased STAT3 protein in enriched ubiquitylated proteins fraction of β5i/LMP7 deficient microglia (**Figure 4A**), suggesting that STAT3 is preferentially degraded by IP in microglia. STAT3 requires phosphorylation to translocate into the nucleus and induce its target genes (32). Indeed, our data indicate that STAT3 nuclear translocation is activated by phosphorylation in β5i/LMP7 KO microglia (**Figures 4A**, **D** and **Supplementary Figure 7A**). Given the role of STAT3 signaling in immunity (33, 34), β5i/LMP7 KO microglia were next investigated for their inflammatory status by assessing the NF-κB pathway. Under basal conditions, NF-κB is located in the cytosol, bound to inhibitor kappa B alpha (IκBα) and stays inactive. Upon pathogen stimuli, IκBα is phosphorylated by its kinase IKK (IκB kinase) and this is sensed by E3 ubiquitin ligase SCF^TRCP^ (35). Phosphorylated IκBα is ubiquitylated and subsequently degraded by the proteasome to release NF-κB for nuclear translocation (36, 37). Western blot analysis showed that NF-κB, IκB and phosphorylated-IκB are increased in IP-deficient microglia (**Figure 4E** and **Supplementary Figure 7B**) in comparison to wild-type. To demonstrate NF-κB activation, we performed fluorescence microscopy and observed increased fluorescence of NF-κB in β5i/LMP7 KO microglia and its translocation to the nucleus (**Figures 4H, I**). In line with this, we observed that NF-κB target cytokine interleukin-6 (IL-6) mRNA level as well as protein secretion were induced in IP-deficient microglia as indicated by qRT-PCR analysis and ELISA, respectively (**Figures 4F, G**). Taken together, these data indicate that β5i/LMP7 deficiency exerts pro-inflammatory effects in primary microglia by chronic NF-κB activation, most likely *via* the stabilisation of STAT3 which is known to exacerbate NF-κB signaling *via* a positive feedback loop (34).

### β5i/LMP7 impairment induces type I interferon response in human microglia

**Figure 5:**
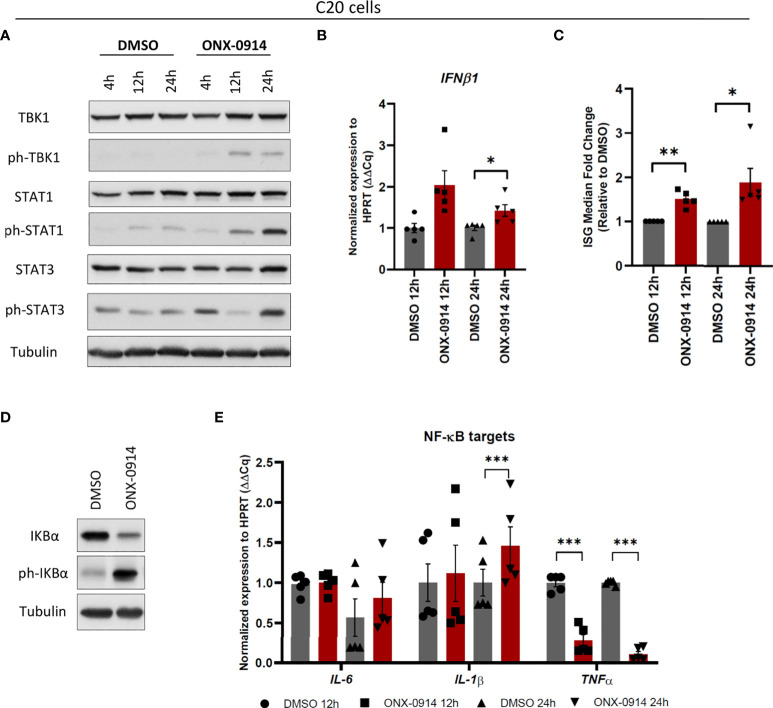
β5i/LMP7 activity inhibition controls activation of innate immune responses in C20 cells. **(A)** Western blot analysis of type I IFN response proteins in C20 cells treated with 200nM of ONX-0914 for indicated time (n=3). **(B, C)** Quantitative reverse transcription PCR (qRT-PCR) analysis of *IFNβ1* **(B)**, and IFN score **(C)** calculated by interferon stimulated genes (ISGs) median fold change in C20 cells treated with 200nM of ONX-0914 for indicated time (n=5). **(D)** Western blot analysis of NF-κB pathway proteins in C20 cells treated with 200nM of ONX-0914 for 24h (n=3). **(E)** qRT-PCR analysis of NF-κB targets in C20 cells treated with 200nM of ONX-0914 for indicated time (n=5). Mean ± SEM; *: P < 0.05, **: P < 0.01, ***: P < 0.001.

Proteasome dysfunction in patients carrying mutations in proteasome subunits of either SP or IP causes dysregulation of type I IFN signaling and induction of interferon-stimulated genes (ISGs) (22, 23). In addition, we previously showed activation of the type I IFN pathway in microglia treated with bortezomib (24). Based on these observations, we next asked whether there is an activation of a type I IFN response to IP impairment. Induction of type I IFNs require a consecutive activation of TANK-binding kinase 1 (TBK1) and interferon regulatory factor 3 (IRF3) by phosphorylation mechanisms (38). Type I IFNs in turn activate Janus kinase (JAK) – STAT signaling resulting in the induction of ISGs (30). Using a dose-dependent experiment with up to 400nM of ONX-0914 for treatment of human C20 microglia, we observed diminished STAT proteins expressions in β5i/LMP7 inhibited human microglia with 400nM of ONX-0914 (**Supplementary Figure 8**) that may be due to induction of cell death. We have determined activation of UPR and Type I IFN response after 200nM of ONX-0914 treatment in C20 cells where cells were still viable (**Supplementary Figures 8** and **2D**). Therefore, this concentration was used for further experiments. As shown in **Figures 5A–C**, β5i/LMP7 inhibition in microglia efficiently engaged a type I IFN response, as indicated by phosphorylation of TBK1 and STAT proteins (**Figure 5A** and **Supplementary Figure 9A**) as well as induction of IFNβ1 (**Figure 5B**) and ISGs (**Supplementary Figure 9B**). Indeed, calculation of IFN score representing the ISG median fold change (39) and it was increased in β5i/LMP7 inhibited human microglia (**Figure 5C**). In contrast to previously published data with bortezomib treated primary mouse microglia (24), β5i/LMP7 KO primary microglia failed to induce a significant type I IFN response (data not shown). Importantly, β5i/LMP7 inhibition in C20 cells was associated with increased phosphorylation and degradation of IκB (**Figure 5D** and **Supplementary Figure 9C**). However, there was a differential impact on NF-κB target genes e.g. *IL-6* (no significant change), *IL-1β* (significantly induced) and *TNF-α* (significantly reduced) following ONX-0914 treatment (**Figure 5E**).

### Protein kinase R senses β5i/LMP7 inhibition by ONX-0914 to trigger autoinflammation in C20 cells

**Figure 6:**
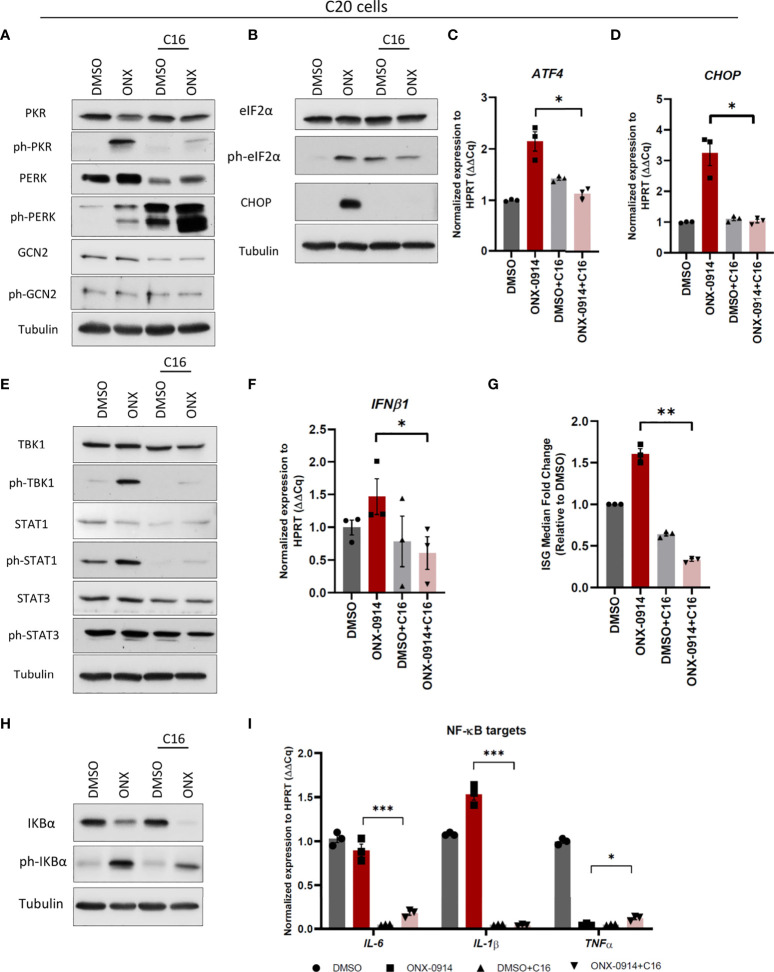
Protein kinase R (PKR) activation controls type I IFN response and NF-κB pathway activation in C20 cells following ONX-0914 treatment. C20 cells were subjected to treatment with 200nM ONX-0914 or DMSO control for 24h following pre-treatment with 1µM PKR inhibitor C16 for 2h. **(A, B)** Western blot analysis of UPR/ISR proteins in C20 cells following treatments. **(C, D)** Quantitative reverse transcription PCR (RT-PCR) analysis of *ATF4* **(C)** and *CHOP* **(D)** mRNA levels in C20 cells following treatments. **(E)** Western blot analysis of type I IFN response proteins in C20 cells following treatments. **(F)** Quantitative RT-PCR analysis of *IFNβ1* mRNA level in C20 cells following treatments. **(G)** IFN score calculated by ISG median fold change in C20 cells following treatments. **(H)** Western blot analysis of NF-κB pathway proteins in C20 cells following treatments. **(I)** Quantitative reverse transcription PCR (RT-PCR) analysis of NF-κB targets mRNA levels in C20 cells following treatments. All data are given (n=3) by mean ± SEM; *: P < 0.05, **: P < 0.01, ***: P < 0.001.

We next tried to unravel the activation mechanisms of immune signaling in human microglia C20 following β5i/LMP7 inhibition. Based on our observation that both PERK and PKR branches of proteotoxic stress response activation in β5i/LMP7 inhibited C20 cells, we subjected these cells to pre-treatments with PERK and PKR specific inhibitors GSK2606414 and C16, respectively (**Supplementary Figure 10A**). C16 treatment substantially attenuated phosphorylation of eIF2α and activation of downstream targets in ONX-0914 treated C20 cells (**Figures 6A–D**). Cytotoxicity of C16 was determined and, the data showed that C20 cell viability slightly decreased after 24h but, ~90% of the C20 cells were still viable (**Supplementary Figure 10B**). Remarkably, as a compensatory mechanism to PKR inhibition, microglia over-activated PERK by phosphorylation, whereas GCN2 phosphorylation was not affected (**Figure 6A**). Most importantly, C16 significantly mitigated the innate immune signaling cascades normally triggered by ONX-0914 (**Figures 6E–G** and **Supplementary Figures 10, 11**), whereas PERK inhibitor treatment failed to influence type I IFN response (**Supplementary Figure 10A**). As shown in **Figures 6E–G**, IP impaired microglia with pre-treatment of C16 showed less TBK1 and STAT proteins phosphorylation as well as decreased levels of IFNβ1, ISGs and IFN score. Moreover, PKR inhibition caused decreased levels of IKBα and phosphorylated IKBα (**Figure 6H**), significant reduction of *IL-6* and *IL-1β* whereas induced *TNF-α* (**Figure 6I**). Collectively, the data show that PKR controls inflammation and drives type I IFN response induced by IP impairment in C20 microglia.

### β5i/LMP7 deficient mice exhibit altered expression levels of microglial markers

**Figure 7:**
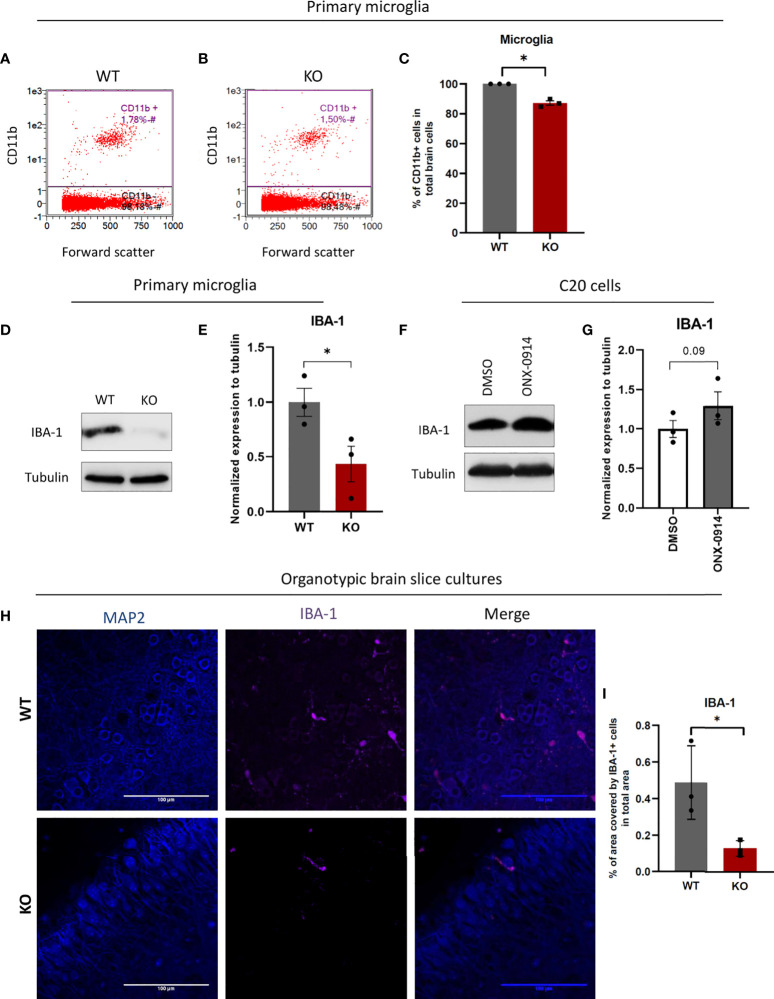
β5i/LMP7 impairment results in less microglia population in mice brains and differs expression levels of microglial surface markers. After brain dissociation, cell suspensions were stained with CD11b FACS antibody to determine microglia amount in the brain by flow cytometry. **(A–C)** Flow cytometric analysis of CD11b+ cells in WT **(A)** and β5i/LMP7 KO **(B)** mice brains, and comparison analysis of CD11b+ cells **(C)**. **(D)** Western blot analysis of ionized calcium-binding adapter molecule 1 (IBA-1) in primary microglia. **(E)** Quantification analysis of IBA-1 Western blot data from **(D)**. **(F)** Western blot analysis of IBA-1 in C20 cells treated with 200nM of ONX-0914 for 24h. **(G)** Quantification analysis of IBA-1 Western blot data from **(F)**. **(H)** Immunofluorescence microscopy analysis of MAP2 and IBA-1 in organotypic brain slice cultures from WT and β5i/LMP7 KO mice brains. **(I)** Analysis of % area covered by IBA-1^+^ cells in total area. All data are given (n=3) by mean ± SEM; *: P < 0.05.

The proteomics analysis showed enriched pathways for cell cycle progression and cytoskeleton organization pointing to altered microglia function in IP deficiency. We next assessed the impact of β5i/LMP7 deficiency on microglia differentiation and activation status. Microglial activation leads to morphological changes, increased motility, fast proliferation as well as increased phagocytosis (40). General microglial markers e.g. cluster of differentiation receptors like CD11b, CD45, CD68, ionized calcium-binding adapter molecule 1 (IBA-1) are present constitutively in the resting state, whereas the expression levels are increased during activation (40). CD11b (also known as integrin alpha M) is expressed on the microglia surface and is involved in adhesion processes (40, 41). CD11b is widely used as a convenient microglial marker for their isolation from brain tissue. We took advantage of this microglial marker to determine the microglia population in the mouse brain and also to isolate microglia from the mouse brain. After brain dissociation, we treated cell suspensions either with CD11b FACS antibody to investigate the influence of β5i/LMP7 deficiency on microglia amount in the brain or, with CD11b MACS antibody to isolate microglia. We observed that β5i/LMP7 KO mice exhibited less microglia in comparison to WT as indicated by flow cytometric analysis of CD11b positive cell population (**Figures 7A–C** and **Supplementary Figure 12A**) indicating that IP is involved in differentiation and proliferation processes. Next, we analyzed the CD11b expression level in MACS isolated microglia using CD11b staining in flow cytometry. We confirmed the high purity of CD11b positive cells (above 97%) after MACS isolation (**Supplementary Figures 12B, C**). Nevertheless, β5i/LMP7 KO microglia exhibited less CD11b expression as measured by the median fluorescence intensity (**Supplementary Figure 12D**). We further determined other general microglial markers; CD68 and IBA-1 in isolated primary microglia using flow cytometry or Western blotting and immunofluorescence microscopy, respectively. CD68 (also known as macrosialin) is a general macrophage marker and transmembrane glycoprotein which is related to the lysosomal/endosomal-associated membrane glycoprotein family (40, 42, 43). Upon inflammatory stimulation, CD68 can be internalized to endosomes and, is therefore used as a marker for phagocytosis and lysosomal activity (40). As expected upon IP-impairment cells start to compensate with lysosomal degradation (more CD68 intensity; **Supplementary Figures 13A, B**), most likely autophagy (44). IBA-1 is a cytosolic microglia marker which is important for motility and migration (40, 45). β5i/LMP7 KO microglia presented less IBA-1 protein expression (**Figure 7D, E**). Furthermore, we detected less IBA-1 positive cells in organotypic brain slice cultures (**Figures 7H, I**). We hereby showed that β5i/LMP7 deficiency causes alterations of microglial function in terms of activation markers for motility and cell numbers. Next, we sought to determine IBA-1 expression in human microglia C20 cells. We observed increased expression of IBA-1 in ONX-0914 treated C20 cells comparison to control DMSO treated cells (**Figures 7F, G**).

### β5i/LMP7-deficient primary microglia exhibit increased phagocytic activity despite less activation in response to amyloid-beta

**Figure 8:**
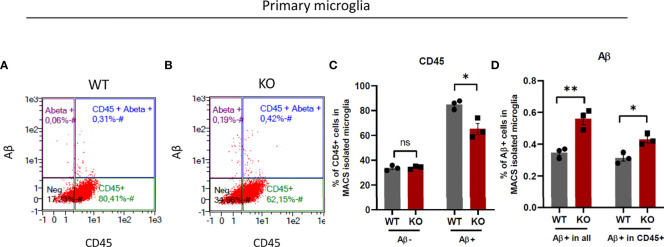
β5i/LMP7 deficient microglia exhibit more phagocytosis activity. **(A–D)** Phagocytosis activity was determined by the analysis of TAMRA-Aβ+ cell population in primary micrgolia. **(A, B)** Gating strategy of flow cytometry analysis of MACS isolated WT **(A)** and β5i/LMP7 KO **(B)** microglia incubated with 2µM TAMRA-labeled amyloid-beta (Aβ) toxic oligomers. **(C)** Bar graphs showing quantification of total CD45+ cells before Aβ treatment, total CD45+ cells after Aβ treatment, **(D)** Bar graphs showing quantification of total Aβ+ cells and Aβ+ cells in CD45+ cells in primary microglia. All data are given (n=3) by mean ± SEM; *: P < 0.05, **: P < 0.01.

Microglia are the resident macrophages of the brain and, as such are responsible for the clearance of apoptotic cells and potentially toxic protein aggregates (1–3). It is suggested that apoptotic cells and protein aggregates activate microglia to be phagocytosed by them (46). CD45 is a macrophage marker expressed low in resting microglia and is used for discrimination of microglia from peripheral infiltrating macrophages which express high CD45 (47). However, recent studies showed that microglia upregulate CD45 in activated state (48, 49) and CD45 high cells are more phagocytic (50). Here, we undertook a comparative examination of microglia isolated from WT and β5i/LMP7 KO mice for their ability to engulf amyloid-beta (Aβ). We incubated WT and β5i/LMP7 KO microglia with fluorophore labelled Aβ for 1 hour, collected the cells, stained them with CD45 and performed flow cytometry (**Figure 8** and **Supplementary Figures 13C, D**). Interestingly, incubating primary microglia with toxic Aβ resulted in CD45 upregulation, which was significantly less pronounced for β5i/LMP7 KO cells (**Figures 8A–C**). Nevertheless, the proportion of Aβ+ cells among CD45+ cells was higher in β5i/LMP7 KO microglia than in the wild-type (**Figure 8D**). From these results we conclude that β5i/LMP7 KO microglia are pre-activated by chronic inflammatory signaling.

## Discussion

By clearing damaged, misfolded, old and/or unneeded proteins, the UPS maintains a stable and healthy intracellular proteome (9, 17). This is particularly important in the CNS where uncontrolled protein accumulation may lead to neuroinflammation and subsequent neurodegeneration (5, 7, 8). Over the last couple of years, microglia function has gained increasing attention for its potential role in triggering neuroinflammation in response to protein aggregation in neurodegenerative diseases (3, 51). Our current understanding is that microglia regulate intracellular protein homeostasis *via* constitutive expression of SP and IP (52–54). Nonetheless, the precise contribution of IP activity on the microglial cellular function and innate immunity of CNS remained unclear. In this report, we show that IP preserves protein homeostasis in microglia and, IP deficiency triggers innate immune responses. In conditions of IP dysfunction, we confirmed impaired proteostasis with accumulation of ubiquitin modified proteins and decreased proteasome active sites accompanied by activation of UPR and ISR (**Figures 1**, **2** and **Supplementary Figure 1-4**). Importantly, the role of UPR and ISR in neuroinflammation has been increasingly realized (55–59). In line with this, loss of function mutations in the genes encoding proteasome components or their assembly factors have been found in patients with rare proteasomopathies suffering from systemic inflammation and/or neurodevelopmental delay (60–64). These proteasomopathies share similar molecular characteristics with proteotoxic stress, activation of UPR and ISR as well as the induction of type I IFN signaling (23, 65, 66). Our previous data also supported the contribution of the IRE1α-arm of the UPR to type I IFN signaling driven by pan-proteasome impairment in microglia (24).

There is increasing evidence showing the importance of PKR in the CNS where it is linked to protein accumulation and disturbed protein homeostasis (67, 68). Supporting this notion, our data identify PKR from the ISR as an important molecular switch in autoinflammation in response to protein homeostasis perturbations caused by IP dysfunction. IP inhibition by ONX-0914 in human microglia triggers both UPR and ISR, as evidenced by increased phosphorylation of PERK and PKR, respectively (**Figure 2**). Accordingly, this is accompanied by sterile induction of type I FN responses and to a lesser extent NF-κB-activated pro-inflammatory cytokines (**Figure 5** and **Supplementary Figures 8, 9**). The data indicate that PKR controls the induction of sterile inflammation driven by IP impairment (**Figure 6** and **Supplementary Figure 10, 11**). Murine microglia lacking IP however display neither PKR activation (**Figure 2**) nor a significant type I IFN response (data not shown). These observations infer that increased protein expression of β5 subunit of SP (**Figure 1**) may protect microglia from type I IFN mediated inflammatory signaling in mouse CNS upon genetic depletion of β5i/LMP7. Indeed, treatment of primary murine microglia with bortezomib targeting both β5 and β5i/LMP7 induces type I IFN signaling depending on UPR (24). Additively, ONX-0914 treated wild-type primary murine microglia activate UPR, however, IP inhibition does not induce type I IFN response in the same settings as reported (24). These results indicate that only immunoproteasome inhibition by ONX-0914 is not enough to induce type I IFN response in primary mouse microglia. Davidson et al. demonstrate that PKR drives induction of type I IFN response in proteasome associated autoinflammatory syndromes depending on its cytosolic interactor IL-24 accumulation (69). We suggest that PKR may drive induction of type I IFN signaling in IP impaired human microglia depending on IL-24 accumulation. It is likely that the mouse microglia model does not develop a type I IFN activation may be due to insufficient IL-24 expression levels (**Supplementary Figure 14**), thereby rendering these cells incapable of inducing PKR activation even under proteotoxic stress conditions. Instead, they activate NF-κB signaling and IL-6 expression. Given the constitutive phosphorylation of eIF2α in wild-type primary microglia (**Figure 2**), it is also conceivable that these cells have already integrated multiple stress signals due to the cell isolation process. Although NF-κB inhibitor IκBα is less efficiently degraded in β5i/LMP7 KO primary microglia model, β5i/LMP7 KO microglia induce NF-κB target *Il-6* mRNA expression by stabilizing NF-κB as a potential IP target (**Figure 4**). This data is in line with other observations found in proteasome impairment settings where proteasome inhibition is accompanied by induction of pro-inflammatory cytokines due to atypical NF-κB signaling (70). Our data indicating activated STAT3 may support the induction of *Il-6* mRNA *via* contributing to stabilisation of NF-κB in the nucleus, leading to chronic activation of NF-κB as shown in cancer models (33, 34). Furthermore, importance of PKR has been described in LPS induced neuroinflammation model in mice as indicated by attenuated microglial immunoreactivity and STAT3 activation as well as less production of amyloid-beta in PKR deficiency (68). Consequentially, imbalanced cellular protein homeostasis and neuroinflammation are the central hallmarks of many neurodegenerative diseases. Other researchers have shown that IP deficiency manipulates pro-inflammatory signaling pathways in murine Alzheimer’s disease models (52, 71). All in all, we conclude that IP control the activation of innate immune pathways *via* proteotoxic stress sensors in microglia.

The UPS is involved in many cellular processes by controlling the abundance of regulatory proteins e.g. transcription factors and enzymes (9–12). Under stress conditions, non-immune cells can induce IP to enhance the proteolytic capacity of the UPS (12, 13). However, immune cells exhibit constitutive expression of IP as well as SP, thus forcing the idea of preferential degradation of cellular proteins by IP in immune cells under basal as well as stress conditions. Our data identifying accumulated ubiquitin-modified proteins in IP deficiency (**Figure 1** and **Supplementary Figure 5**) and identification of these ubiquitin-modified proteins by proteomics (**Figure 3** and **Supplementary Figure 6**) prove our hypothesis on the importance of IP in microglia function. Accordingly, IP deficiency alters the profile of ubiquitylated proteins mostly seen in the vital mechanisms of microglial function including cytoskeletal organization, cell cycle, ribosomal function and, accumulates the ubiquitylated proteins involved in these mechanisms as well as energy metabolism and immune responses of microglia at steady state and/or under challenged conditions (**Figure 3** and **Supplementary Figure 6**). In support, the decreased microglia cell count indicates IP-deficient mice present affected microglial cell differentiation/proliferation processes in line with altered levels of cell cycle regulatory proteins e.g. COP9 signalosome subunit3 (Cops3), Ras homolog enriched in brain (Rheb) and Myristoylated alanine-rich C-kinase substrate (Marcks) (**Figure 3D**). Cops3 is a component of COP9 signalosome complex which is involved in the regulation of cell cycle progression (72). Rheb is a GTP-binding protein with a function of cell cycle regulation (73). Marcks is not only involved in the regulation of cell cycle but also cell motility and neural development (74). Furthermore, IP-deficient microglia slightly change the expression of activation markers (**Figure 7** and **Supplementary Figures 12, 13**), thus disturbing of microglial function, since their function is closely linked to their activation. IP-deficient microglia display impaired motility and adhesion markers IBA-1 and CD11b (40, 41, 45), respectively (**Figure 7** and **Supplementary Figure 12**), whereas slightly increase lysosomal/endosomal-associated transmembrane protein CD68 (**Supplementary Figure 13**). It has been shown that CD11b-deficient microglia display chronic NF-κB activation and CD11b expression is linked to TLR activation-induced signaling (75). CD68 is involved in phagocytosis and can be induced during inflammation (40, 42, 43). As illustrated in **Figure 3**, IP deficiency affects cytoskeleton organization and endosomal pathway proteins which can impair the motility and phagocytotic functions of microglia, respectively. It has been shown that microglia with low IBA-1 and high CD68 expression are related to neurodegenerative diseases and aging (76–78). Microglia play an important role in the control of age-associated neurodegenerative diseases (51). Conversely, IBA-1 expression is slightly increased IP inhibited human microglia C20 cells (**Figure 7**). We suggest that increased activation marker in C20 cells upon IP inhibition may correlate with the activated type I IFN signaling (**Figure 5**) (79, 80).

Protein aggregates including pathologic Aβ fibrils from the milieu can be sensed by pattern-recognition in microglia, which in turn are activated to eliminate them (3). Upon exposure to toxic Aβ oligomers, IP-deficient microglia incapacitate activation as indicated by less CD45 (**Figure 8**). Despite decreased response to toxic Aβ, IP-deficient microglia preserve more phagocytosis activity. This observation may seem surprising at first sight, since it would imply that microglia devoid of IP protect the CNS from extracellular Aβ peptide depositions and plaques. This interpretation is also in conflict with an earlier study showing Aβ-plaque pathology is not affected by β5i/LMP7 deficiency in Alzheimer’s disease (AD)-like APPPS1 mice (71). However, our data do not suggest that Aβ aggregates are efficiently degraded by β5i/LMP7 KO microglia following ingestion and, further investigations will be required to address this issue.

In conclusion our findings highlight that IP impairment alters microglial function, induces proteotoxic stress, and promotes sterile activation of innate immune signaling pathways in microglia.

## Data availability statement

The datasets presented in this study can be found in online repositories. The names of the repository/repositories and accession number(s) can be found below: https://www.ebi.ac.uk/pride/archive/, PXD034900.

## Author contributions

GÇ, MS-T and EK discussed and developed the study concept. GÇ designed the experiments along with input from MS-T, FE and EK. GÇ performed the experiments and analyzed the data. GÇ, MS-T and HJ performed the animal experiments. GÇ and SV performed microscopy experiments with primary microglia. ES performed microscopy experiments with organotypic brain slice cultures. GÇ and SV performed sample preparation for proteomic study and data analysis. EH and UV performed LC/MS-MS measurements and participated in proteomic data analysis. GÇ, FE and EK wrote the manuscript along with input from MS-T. All authors contributed to the article and approved the submitted version.

## Funding

This research was supported by the German Research Foundation (CRC/TRR 167), SFB/TRR 167 (to GÇ), Research Training Group 2719 RTG-PRO (to FE and EK).

## Acknowledgments

We acknowledge Dr. Vladimir Milenkovic (Department of Psychiatry and Psychotherapy, University of Regensburg) for kindly providing C20 cells. For excellent assistance, we wish to thank Dr. Vishnu Mukund Dhople (Interfaculty Institute of Genetics and Functional Genomics, University Medicine Greifswald) for operation of mass spectrometers. We are further grateful to the animal facility for support with the mouse work performed under the reference number 7221.3-1-074/17-1 (sacrification notification). We acknowledge support for the Article Processing Charge from the DFG (German Research Foundation, 393148499) and the Open Access Publication Fund of the University of Greifswald.

## Conflict of interest

The authors declare that the research was conducted in the absence of any commercial or financial relationships that could be construed as a potential conflict of interest.

## Publisher’s note

All claims expressed in this article are solely those of the authors and do not necessarily represent those of their affiliated organizations, or those of the publisher, the editors and the reviewers. Any product that may be evaluated in this article, or claim that may be made by its manufacturer, is not guaranteed or endorsed by the publisher.
