# Supplementary material for: Immunoproteasomes control activation of innate immune signaling and microglial function
Source: Front Immunol. 2022 Oct 6;13:982786. doi: 10.3389/fimmu.2022.982786 (PMC9584546; doi:10.3389/fimmu.2022.982786)

Immunoproteasomes control activation of innate immune signaling and microglial function

Supplementary Material

Gonca Çetin[1], Maja Studencka-Turski[1], Simone Venz[1], Eileen Schormann[2], Heike Junker[1], Elke Hammer[3], Uwe Völker[3], Frédéric Ebstein[1], Elke Krüger[1]*

[1]Institute of Medical Biochemistry and Molecular Biology, University Medicine Greifswald, Greifswald, Germany

[2]Institute of Biochemistry, Charité – University Medicine Berlin, Berlin, Germany

[3]Interfaculty Institute of Genetics and Functional Genomics, University Medicine Greifswald, Greifswald, Germany

*** Correspondence:**
Elke Krüger
elke.krueger@uni-greifswald.de

Supplementary Table 1

**Primary Antibodies**	**Dilution**
β5i/LPM7 (Serum - for mouse)	1:100000
β5i/LPM7 (sc-365699, Santa Cruz - for human)	1:10000
β1i/LMP2 (ab3328, Abcam)	1:2000
β2i/MECL-1 (Serum)	1:50000
β1 (Serum)	1:200000
β2 (MCP165, EnzoLife Sciences)	1:6000
β5 (ab3330, Abcam)	1:2000
Ubiquitin-K48 (#8081, Cell Signaling)	1:1000
IRF3 (#4302, Cell Signaling)	1:1000
ph-IRF3 (#4947, Cell Signaling)	1:1000
STAT1 (#9172, Cell Signaling)	1:1000
ph-STAT1 (MA5-15071, Thermo Scientific)	1:1000
STAT3 (ab50761, Abcam)	1:500
ph-STAT3 (ab76315, Abcam)	1:1000
TBK1 (#3013, Cell Signaling)	1:1000
ph-TBK1 (#5483, Cell Signaling)	1:1000
IKBα (#4812, Cell Signaling)	1:1000
ph-IKBα (#9246, Cell Signaling)	1:1000
NF-κB p65 (#4764S, Cell Signaling)	1:1000
eIF2α (#7922, Cell Signaling)	1:1000
ph-eIF2α (#7921, Cell Signaling)	1:1000
ATF4 (#11815, Cell Signaling)	1:1000
CHOP (2895S, Cell Signaling)	1:1000
PERK (#3192, Cell Signaling)	1:1000
ph-PERK (#3179, Cell Signaling)	1:1000
PKR (#12297, Cell Signaling)	1:1000
ph-PKR (ab226852, Abcam)	1:4000
GCN2 (#65981, Cell Signaling)	1:1000
ph-GCN2 (AF7605-SP, R&D Systems)	1:1000
IBA-1 (1919741, Wako)	1:2000
Tubulin (ab7291, Abcam)	1:50000
β-actin (sc47778, Santa Cruz)	1:5000
**Secondary Antibodies**	**Dilution**
Anti-rabbit (#7074, Cell Signaling)	1:10000
Anti-mouse (#7076, Cell Signaling)	1:10000

Supplementary Table 2

Gene	Primers for mouse genes (5' → 3')
Hprt	for GATCAGTCAACGGGGGACAT<br>rev ACTTGCGCTCATCTTAGGCT
Stat3	for GACACCATTCATTGATGCAG<br>rev AAACGTGAGCGACTCAAAC
Il-6	for ACCAAGAGATAAGCTGGAGT<br>rev TAGGCATAACGCACTAGGTT
Il-24	for TGAAGAACACTGTGCAAACTCA<br>rev TGCGGAACAGCAAAAACCG
**Gene**	**Primers for human genes (5' → 3')**
IL-24	for CTGATATCTGCAGGGACAGA<br>rev AGAAGGCCTTTTCTAGCTGT

Supplementary Table 3

Gene	TaqMan probes for human genes
HPRT1	Hs99999909_m1
ATF4	Hs00909569_g1
CHOP	Hs00358796_g1
IFNβ1	Hs01077958_s1
IFI44	Hs00951349_m1
ISG15	Hs00192713_m1
IFIT1	Hs00356631_g1
IFI27	Hs01086373_g1
RSAD2	Hs00369813_m1
OASL	Hs00388714_m1
IFI44L	Hs00915292_m1
MX1	Hs00895608_m1
IL-6	Hs00985639_m1
IL-1β	Hs00174097_m1
TNFα	Hs00174128_m1

Supplementary Table 4
Description of experiment settings for LC-MS/MS analysis and database search

Data dependent analyses (DDA)	
reversed phase liquid chromatography	**nano-Acquity UPLC (Waters Corp)**
trap column	nanoACQUITY UPLC Symmetry C18 Trap Column, 100Å, 5 µm, 180 µm x 20 mm, 2G, V/M, Waters
analytical column	BEH C18 nanoACQUITY Column 10K psi, 130Å, 1.7 µm, 100 µm X 100 mm, Waters
flow rate	400 nl/min
column oven temperature	40°C
buffer system	binary buffer system consisting of 0.1% acetic acid in HPLC-grade water (buffer A) and 100% ACN in 0.1% acetic acid (buffer B)
gradient	gradient of buffer B: 5min 1% to 5 %, 63min 5% to 25%, 25min 25 to 60%, 2min 60% to 99%, 2min 99%, 1min 99% to 1%, 5 min 1%
Mass spectrometer	**LTQ Orbitrab Velos**
operation mode	data-dependent
electrospray	Nanospray Ion Source
Full MS	
MS scan resolution	30,000
AGC target	1e6
maximum ion injection time	10 ms
scan range	325 to 1525 *m/z*
spectra data type	profile
dd-MS2	
MS/MS AGC target	3e4
minimum ACG target	-
intensity threshold	2e3
maximum ion injection time for the MS/MS scans	100 ms
number of MS/MS scans	Top 20
spectra data type	centroid
selection for MS/MS	1
isolation window	2.0 Da
fixed first mass	-
dissociation mode	collisional induced dissociation (CID)
normalized collision energy	35
charge exclusion	unassigned,1, >3
dynamic exclusion	60 sec

Proteome Discoverer 2.4	
Analysis Type: DDA	
Serach engine used:	Sequest HT
Min Peptide Length:	7
Max Peptide Length:	144
Missed Cleavages:	3
Digest Type:	Specific
Enzymes / Cleavage Rules:	Trypsin/P
Max Variable Modifications:	5
Database Original File:	mouse_uniprot_swissprot_2020_04.fasta
Fixed Modifications::	
Variable Modifications: :	Carbamidomethyl (C)<br>Oxidation (M)<br>GG (K)<br>LRGG (K)<br>Nethylmaleimide (C)<br>Acetyl (Protein N-term)
Mass tolerance for precursor ions:	10 ppm
Mass tolerance for fragment ions:	0.6 Da
Threshold score peptides	FDR 0.01
Threshold score proteins	FDR 0.01
Threshold score for accepting protein identification	Two peptides (unique + razor)
Software/method used to evaluate site assignment	No PTM reported

Supplementary Figure 1. Quantification analysis of proteasome catalytic subunits in primary microglia, gating strategy of flow cytometry analysis from Figure 1 and fluorescence microscopy analysis of organotypic brain slice cultures. (A) Quantification analysis of Western blot data from Figure 1A-B**. (B)** Gating strategy for primary microglia. Cells were determined by forward scatter area and side scatter area. Singlets were selected and cells were gated by negative control. **(C), (D)** Fluorescence microscopy analysis of organotypic brain slice cultures stained with pan-reactive proteasome activity-based probe Me4BodiPyFL-Ahx3Leu3VS (ABP) (488nm) (B) and, quantification of the microscopy data. All data are given (n=3) by mean ± SEM; *: P<0.05, **: P < 0.01, ***: P < 0.001.

Supplementary Figure 2. Quantification analysis of proteasome catalytic subunits in C20 cells and gating strategy of flow cytometry analysis from Figure 1, and cytotoxicity of ONX-0914 on C20 cells. (A), (B) Quantification analysis of immunoproteasome (A) and standard proteasome (B) catalytic subunits Western blot data from Figure 1E. **(C)** Gating strategy of flow cytometry analysis for C20 cells in Figure 1. **(D)** Cell viability analysis of C20 cells following ONX-0914 treatment with the dose of 200nM for indicated time. All data are given (n=3) by mean ± SEM; *: P<0.05.

Supplementary Figure 3. Immunoproteasome impairment disturbs protein homeostasis and activates PERK arm of UPR. (A) Western blot analysis of poly-ubiquitylated proteins in human microglia C20 cells treated with different doses of ONX-0914 for 24h (n=3). **(B)** Quantification analysis of Western blot data from A. **(C)** Western blot analysis of PERK pathway proteins in C20 cells treated with different doses of ONX-0914 for 24h (n=3). **(D)** Quantification analysis of Western blot data from C. **(E)** Western blot analysis of UPR proteins and PCR analysis of transcription factor Xbp1 cDNA in C20 cells treated with 200nM ONX-0914 or DMSO for indicated time. Mean ± SEM; *: P<0.05, ***: P < 0.001.

Supplementary Figure 4. Quantification analysis of Western blot data from Figure 2. (A) Analysis of UPR/ISR proteins in C20 cells treated with 200nM of ONX-0914 or DMSO control for indicated time. **(B)** Analysis of UPR/ISR proteins in primary microglia. All data are given (n=3) by mean ± SEM; *: P<0.05, ***: P < 0.001.

Supplementary Figure 5. Enrichment of ubiquitylated proteins. Ubiquitin-modified proteins were enriched and prepared for mass spectrometric analysis. **(A)** Scheme showing the enrichment process. Protein extracts incubated with Ubi-Qapture-Q matrix containing ubiquitin-binding domain (UBD), ubiquitin-modified proteins were captured by the matrix and eluted, unmodified proteins in flow through were collected as unbound fractions. **(B)** Western blot analysis of poly-ubiquitylated proteins in untreated, 8h 50nM BTZ treated, and 8h 1μg/mL LPS stimulated WT and β5i/LMP7 KO primary microglia total protein extracts. **(C), (D)** Western blot analysis of ubiquitylated proteins in unbound fractions (B) and elution fractions (C) after enrichment assay. **(E)** Quantification analysis of Western blot data from B. Mean ± SEM; ***: P < 0.001.

Supplementary Figure 6. Identification of ubiquitylated proteins by proteomics. (A) Scheme showing the identification process. Enriched ubiquitin-modified proteins were prepared for LC-MS/MS measurement using single pot solid-phase enhanced sample preparation (SP3) technology. Protein identification and quantification were performed using Proteome Discoverer 2.4. **(B)** Venn diagrams showing the total numbers of identified proteins in each experiment (n=3). **(C), (D), (E)** Venn diagrams showing the numbers of identified proteins in untreated (C), 8h 50nM BTZ treated (D) and 8h 1μg/mL LPS stimulated (E) WT and β5i/LMP7 KO primary microglia (n=3; fold change cut off 1,3). **(F)** Table showing the most affected cellular mechanisms (top 5) in immunoproteasome (IP) deficiency as indicated by the change of ubiquitylated proteins in β5i/LMP7 KO comparison to WT (n=3; fold change cut off 1,3). **(G)** Table showing affected cellular mechanisms (top 5), which proteins preferentially degraded by IP involved in (n=3; fold change cut off 1,3).

Supplementary Figure 7. Translocation of phosphorylated STAT3 to nucleus from cytosol and increase of nuclear factor-kappa B (NF-κB) pathway proteins in primary murine microglia. (A) Western blot analysis of ph-STAT3 translocation to nucleus in primary mouse microglia. **(B)** Quantification analysis of ph-STAT3 in nuclear fraction (normalized to Histone3). All data are given (n=3) by mean ± SEM; *: P < 0.05.

Supplementary Figure 8. Analysis of type I IFN response proteins in C20 cells treated with different doses of ONX-0914 for 24h. (A) Western blot analysis of type I IFN response proteins. **(B)** Quantification analysis of Western blot data from A.

Supplementary Figure 9. Analysis of innate immune signaling activation in C20 cells upon IP inhibition. (A) Quantification analysis of Western blot data from Figure 5A (n=3). **(B)** qRT-PCR

analysis of ISGs in ONX-0914 treated C20 cells for indicated time (n=5). **(C)** Quantification analysis of Western blot data from Figure 5D (n=3). Mean ± SEM; *: P<0.05, **: P < 0.01, ***: P < 0.001.

Supplementary Figure 10. ISR inhibitor treatments in C20 human microglia. (A) C20 cells pre-treated with different doses of GSK2606414 or C16 for 2h and subjected to 200nM of ONX-0914 for 24h. **(B)** Cell viability analysis of C20 cells following 1µM C16 treatment for indicated time. **(C)** Quantification analysis of Western blot data from Figure 6A-B. All data are given (n=3) by mean ± SEM; *: P<0.05, **: P < 0.01.

Supplementary Figure 11. PKR controls activation of innate immune signaling in human microglia C20 cells upon IP inhibition. (A) Quantification analysis of Western blot data from Figure 6E. **(B)** qRT-PCR analysis of ISGs in pre-treated with 1µM of C16 and subjected to 200nM of ONX-0914 C20 cells for 24h. **(C)** Quantification analysis of Western blot data from Figure 6H. All data are given (n=3) by mean ± SEM; *: P<0.05, **: P < 0.01, ***: P < 0.001.

Supplementary Figure 12. Flow cytometric analysis and gating strategies for CD11b staining. (A) Gating strategy of flow cytometry analysis of total cells in the brain, singlets were selected and cells were gated by negative control. **(B)** Gating strategy of flow cytometry analysis of MACS isolated CD11b+ cells, singlets were selected and cells were gated by negative control. **(C), (D)** Flow cytometric analysis of MACS isolated CD11b+ WT and β5i/LMP7 KO cells. Mean ± SEM (n=3); **: P<0.01.

Supplementary Figure 13. Flow cytometric analysis and gating strategy for CD68 or CD45 and amyloid beta (Aβ) treated primary microglia. Cells were detected by forward scatter area and side scatter area, singlets were selected and cells were gated by negative control. **(A), (B)** Flow cytometric analysis of CD68+ cells in wild type (WT) and β5i/LMP7 knockout (KO) primary microglia. **(C), (D)** Flow cytometric analysis of CD45+ cells in wild type (WT) and β5i/LMP7 knockout (KO) primary microglia. **(E)** Gating strategy of flow cytometry analysis of Aβ+ cells in CD45+ cells. Mean ± SEM (n=3); *: P<0.05.

Supplementary Figure 14. Transcriptional analysis of IL-24 in microglia. (A) qRT-PCR analysis of IL-24 in C20 cells and primary mouse microglia isolated from WT and β5i/LMP7 KO mice. Data are given (n=3) by mean ± SEM.

Supplementary Figure 1

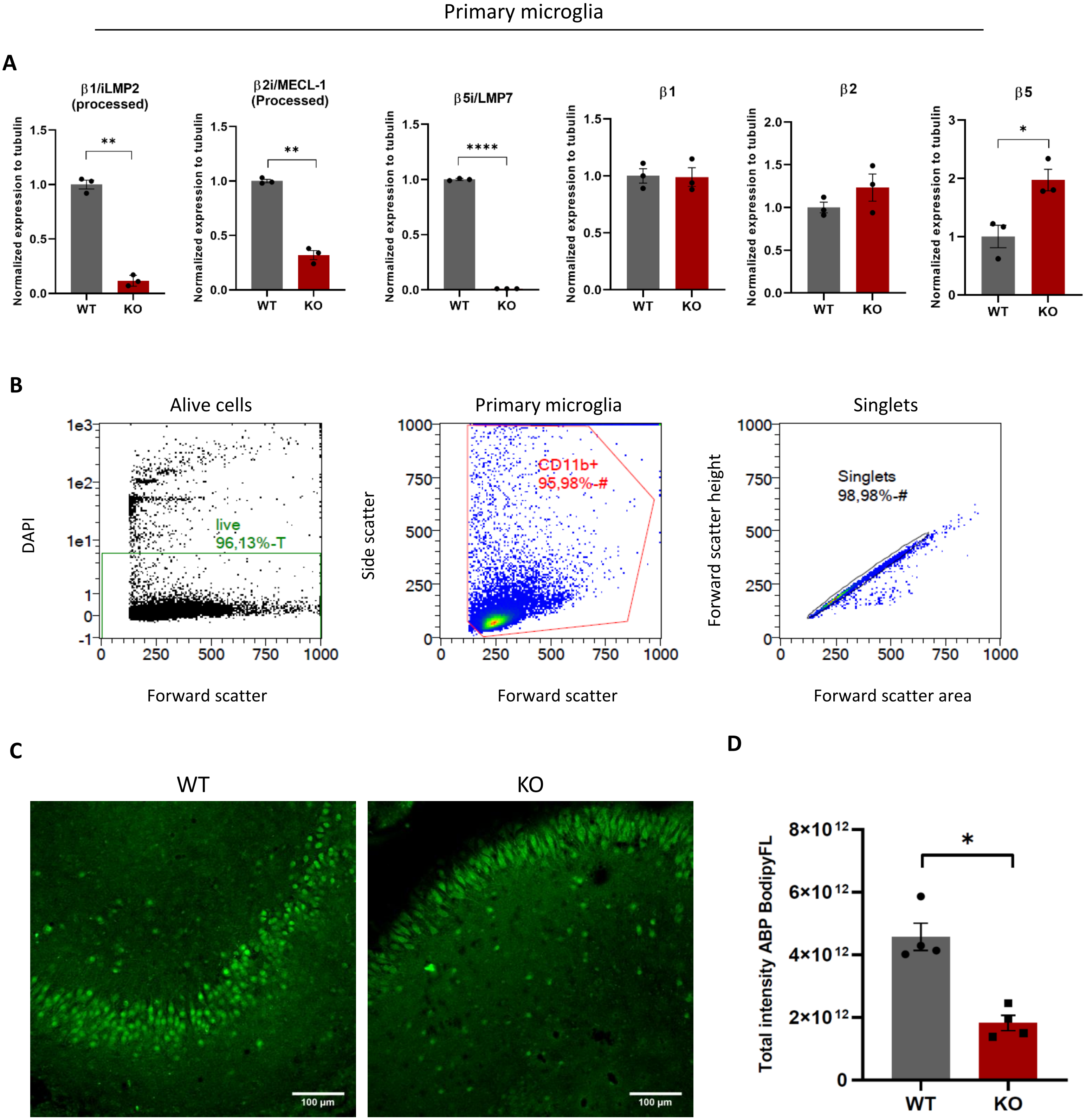

Supplementary Figure 2

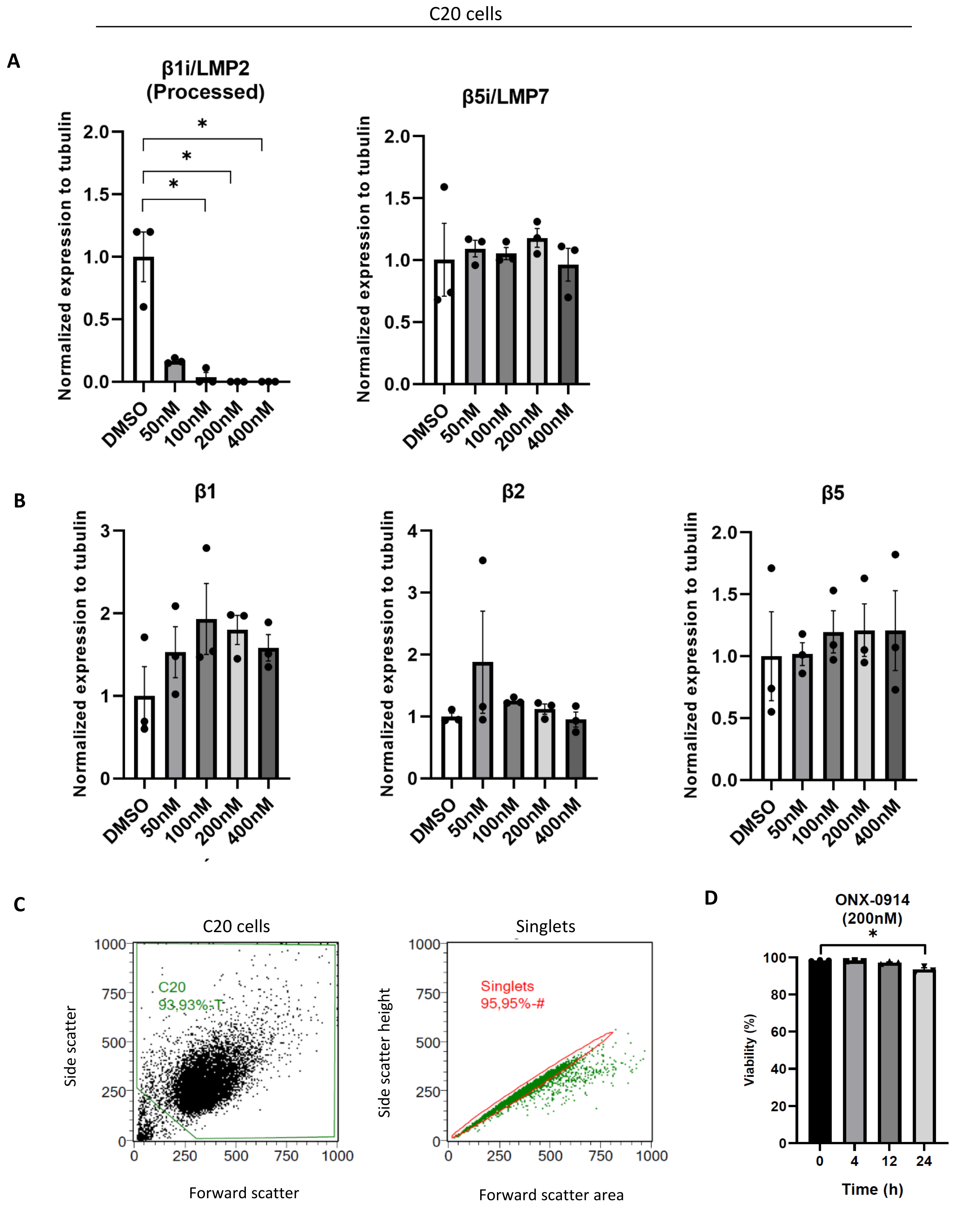

Supplementary Figure 3

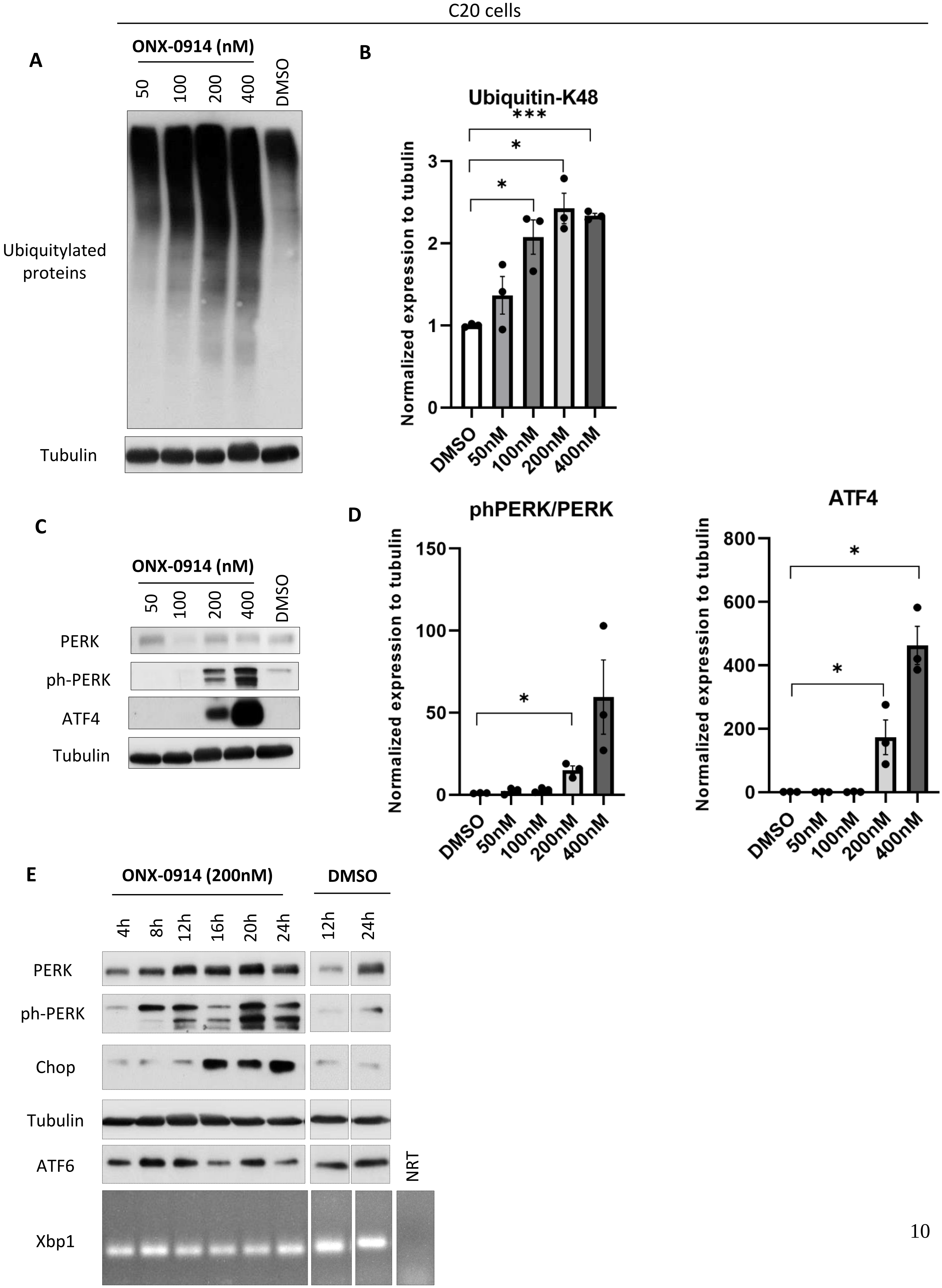

Supplementary Figure 4

C20 cells

A

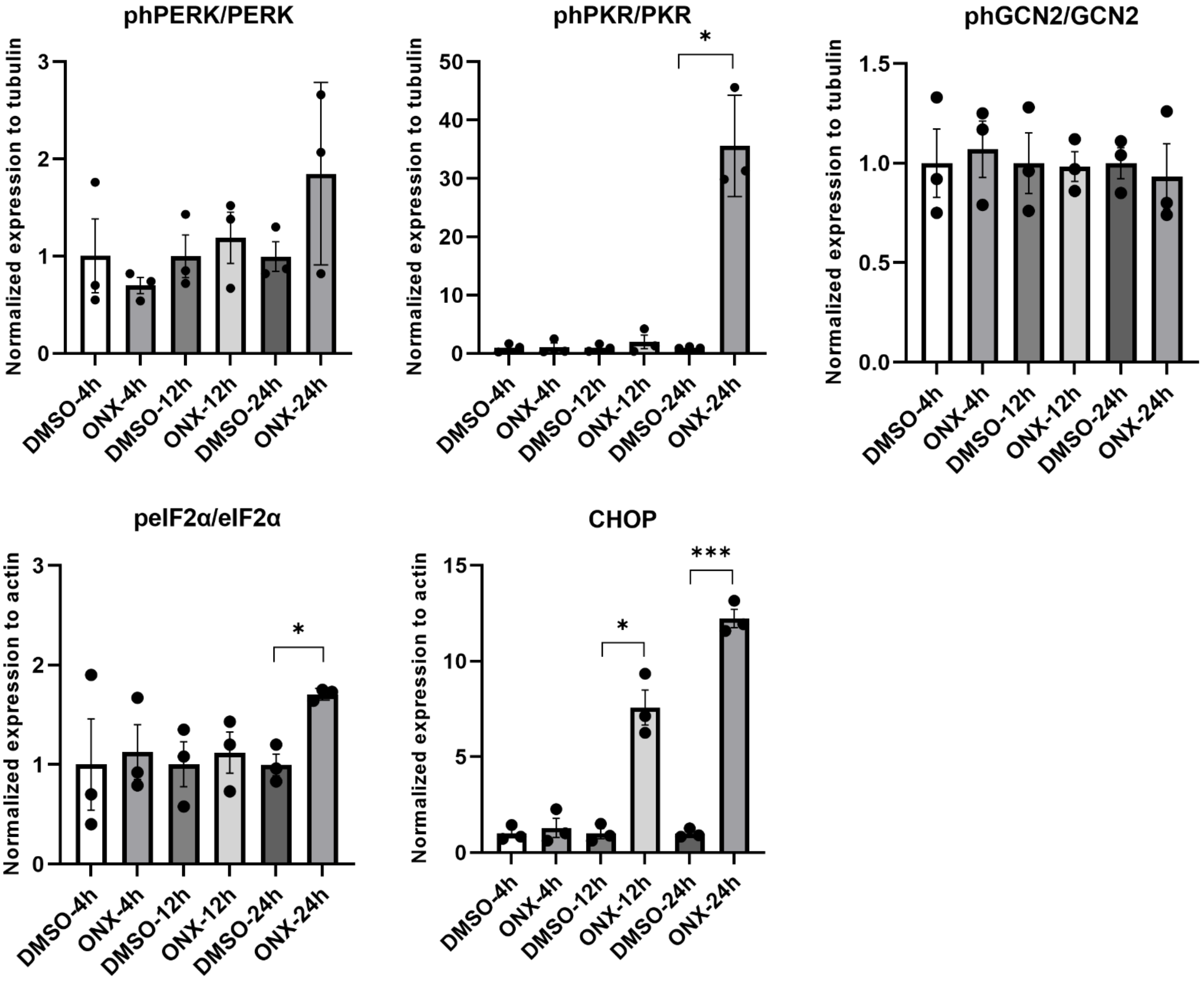

Primary microglia

B

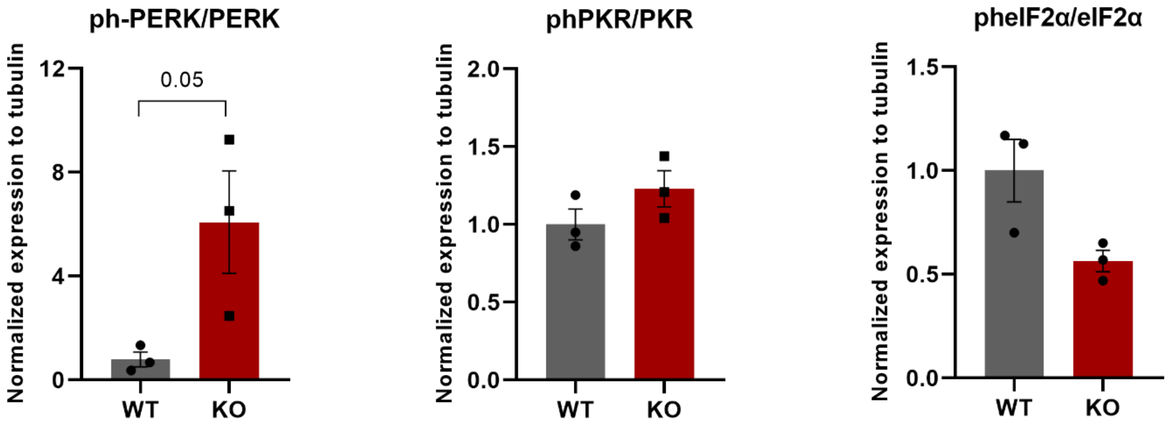

Supplementary Figure 5

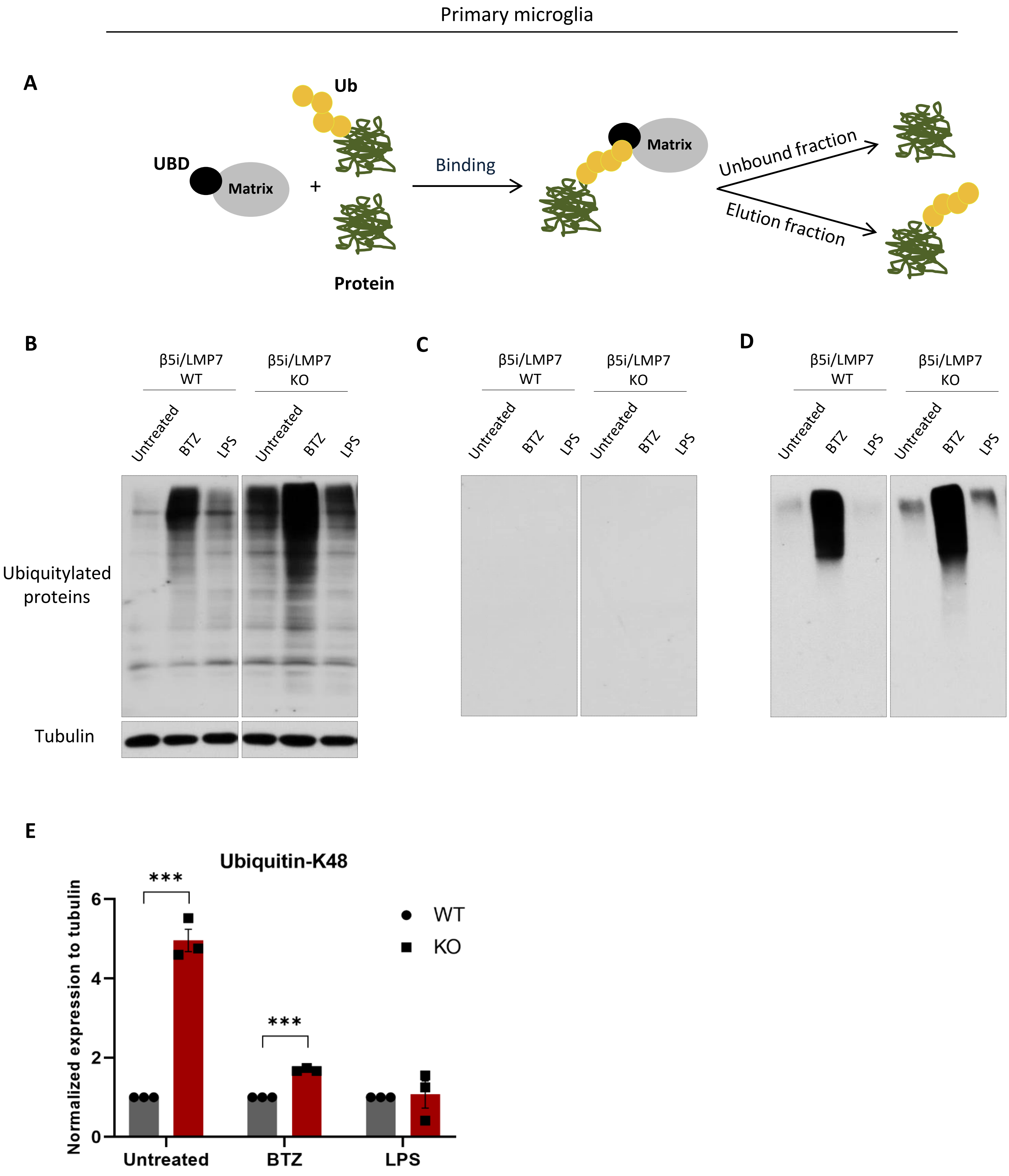

Supplementary Figure 6

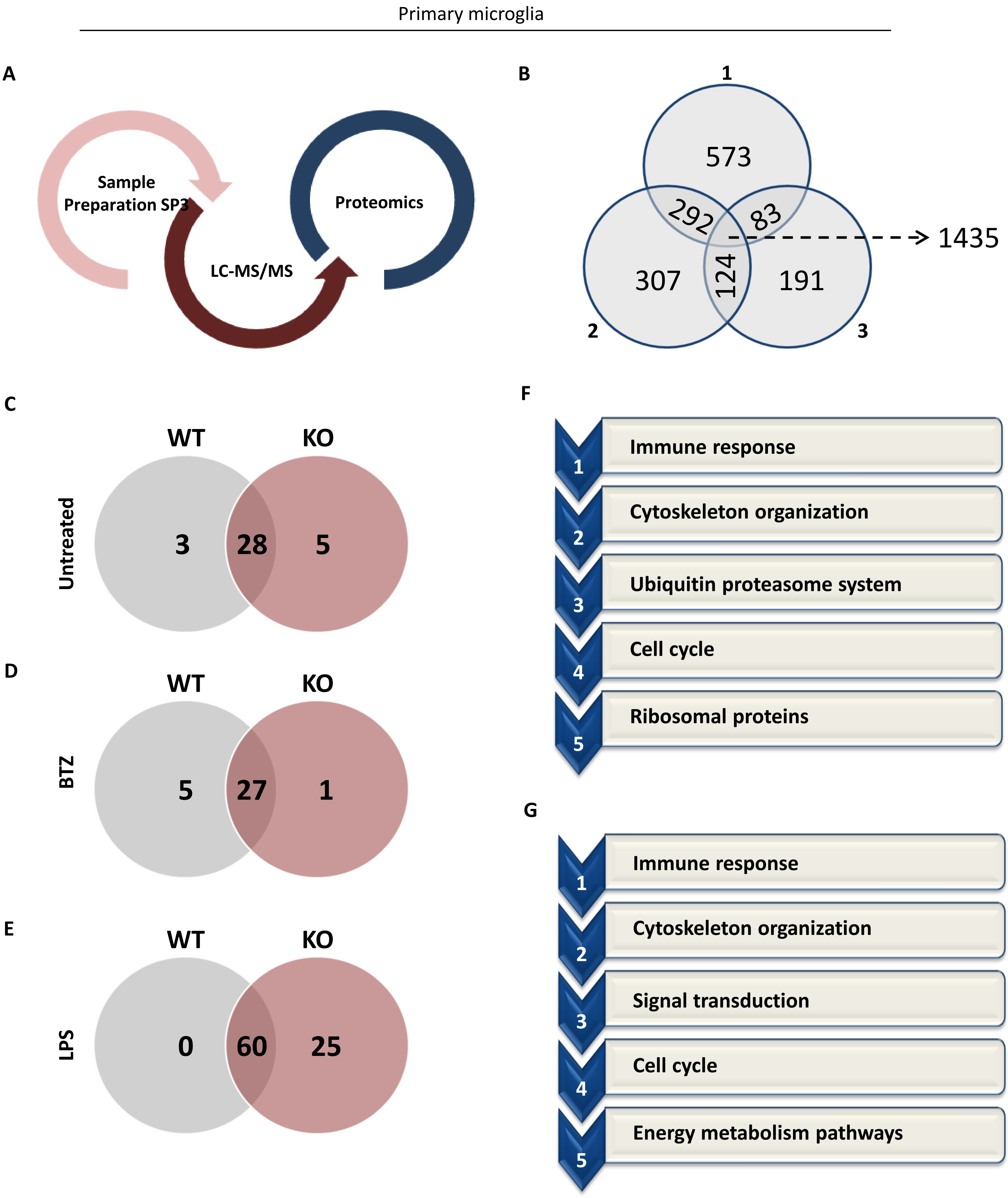

Supplementary Figure 7

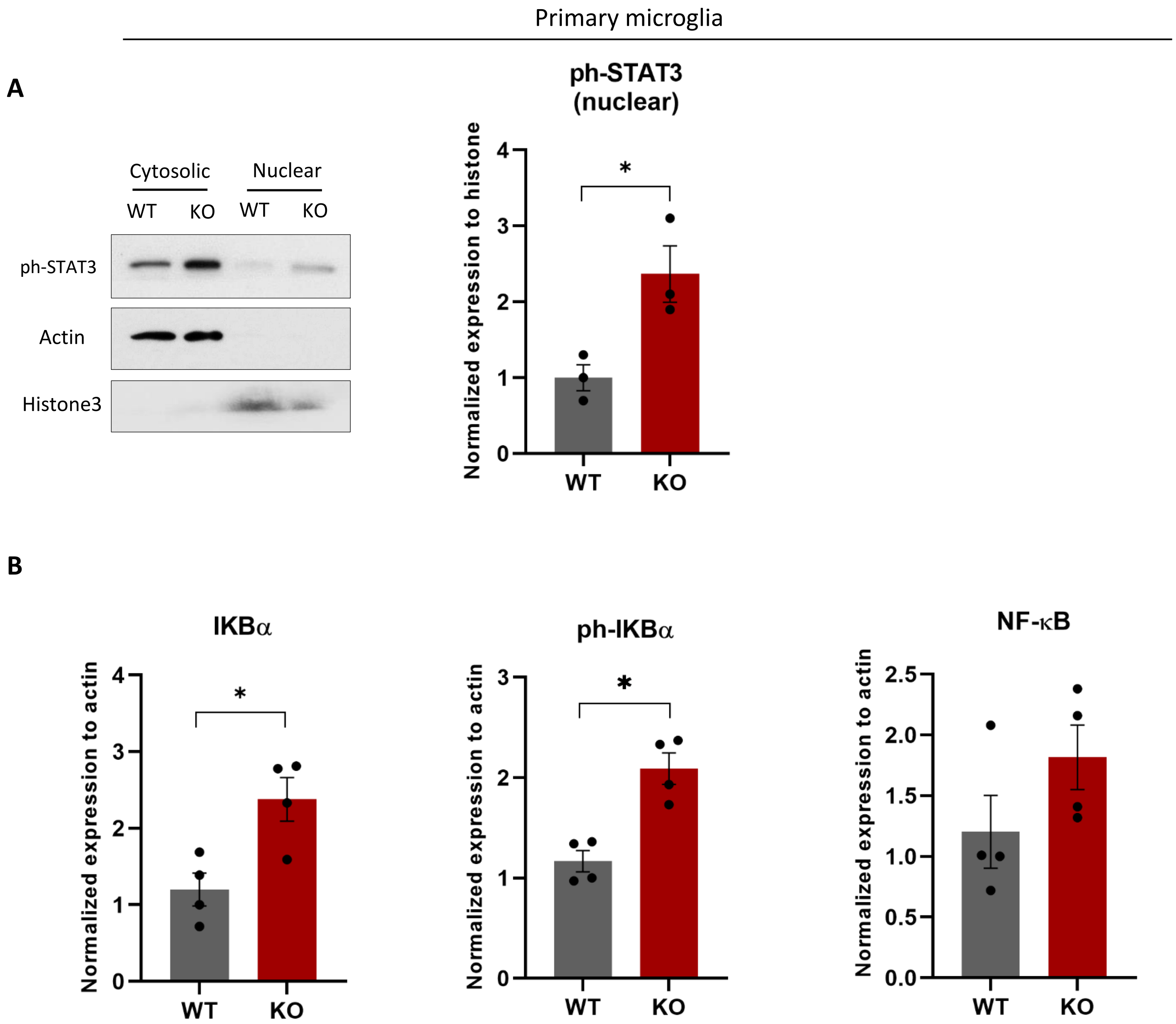

Supplementary Figure 8

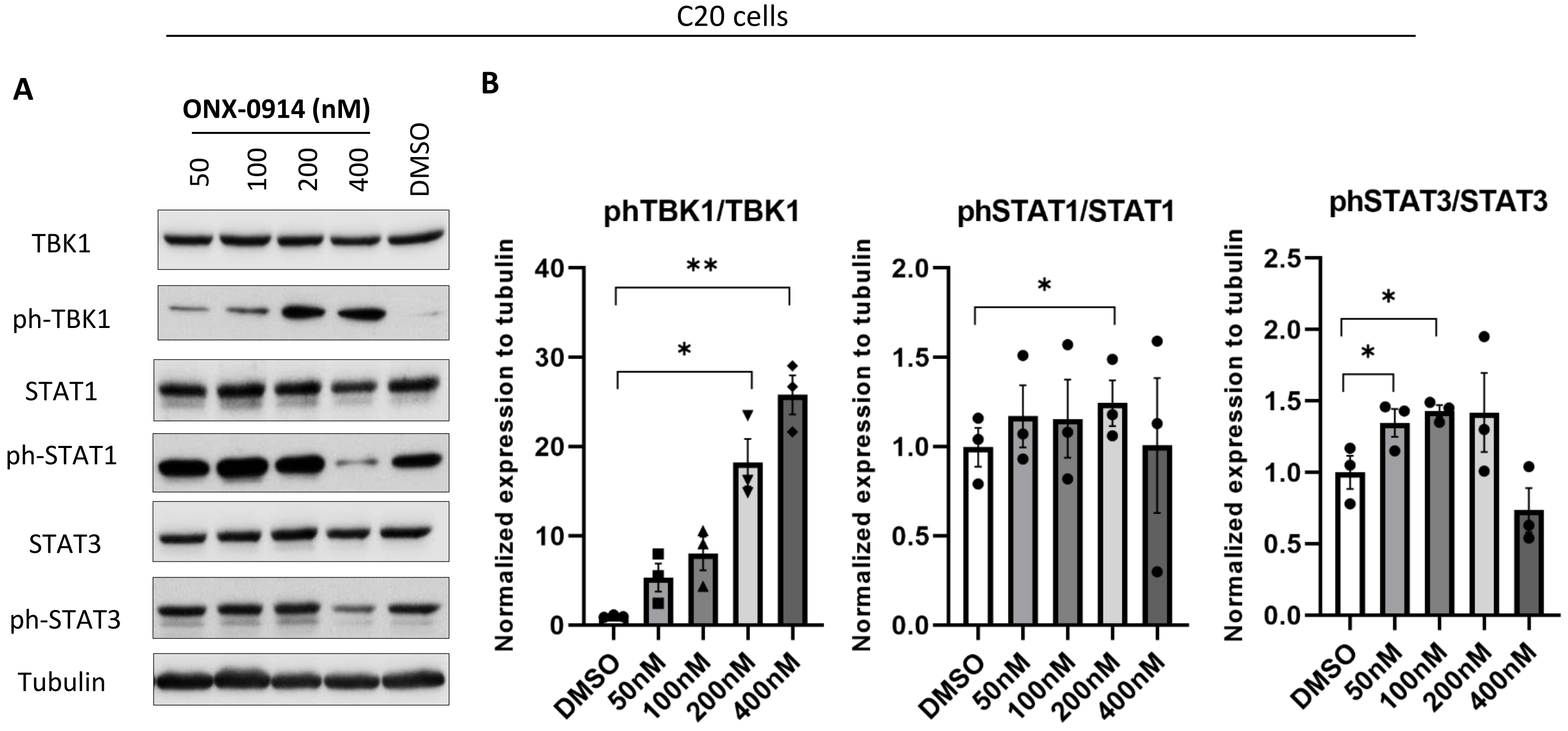

Supplementary Figure 9

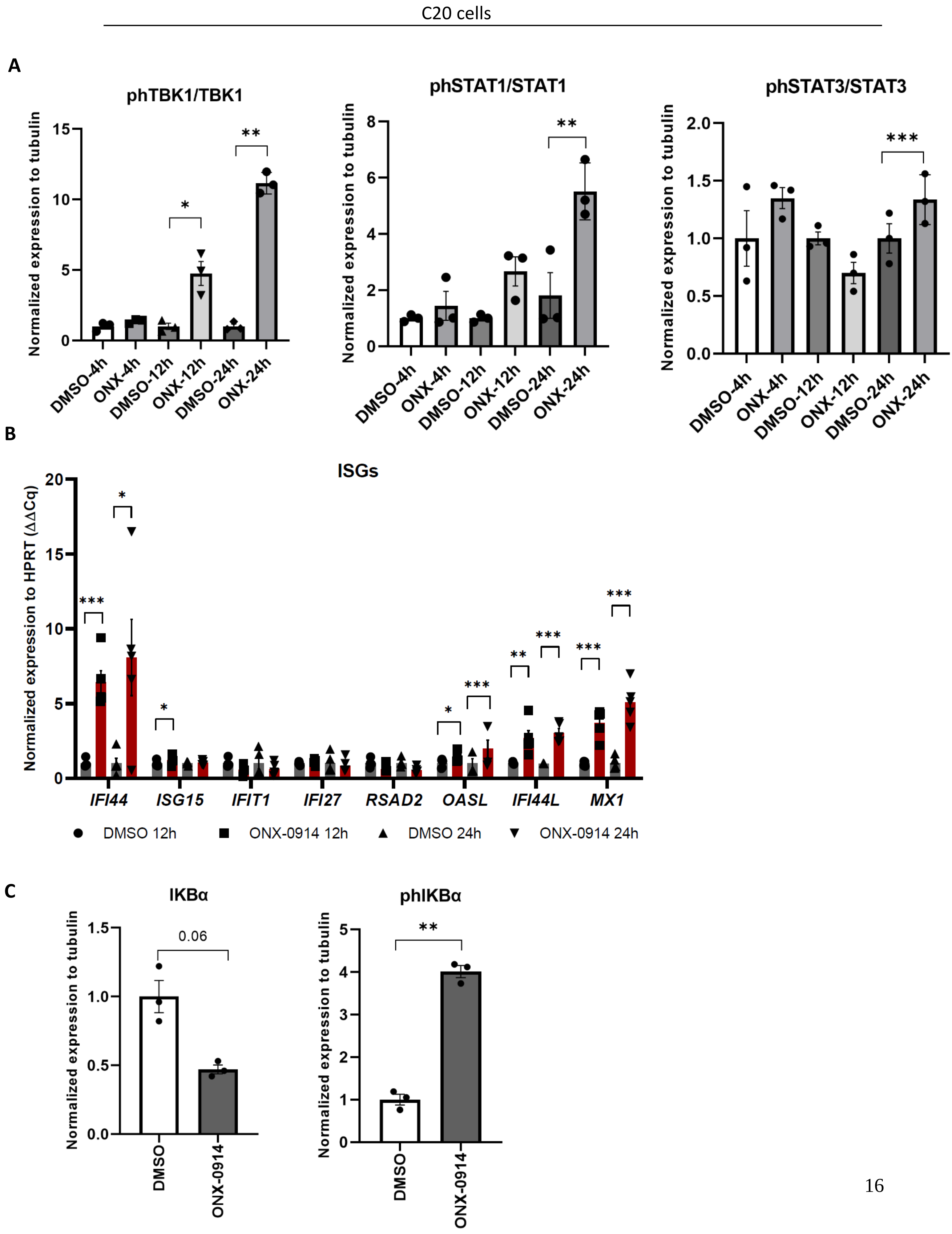

Supplementary Figure 10

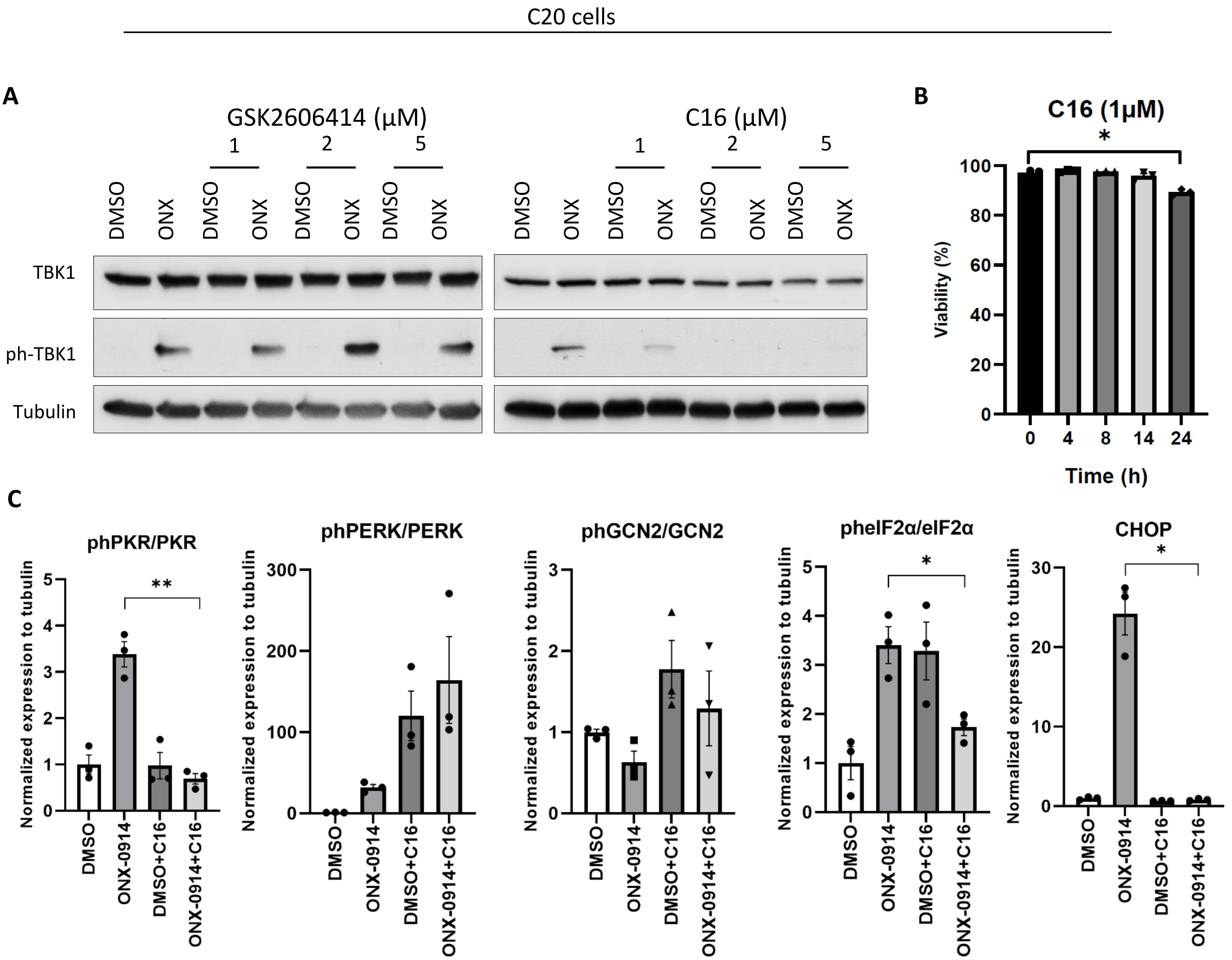

Supplementary Figure 11

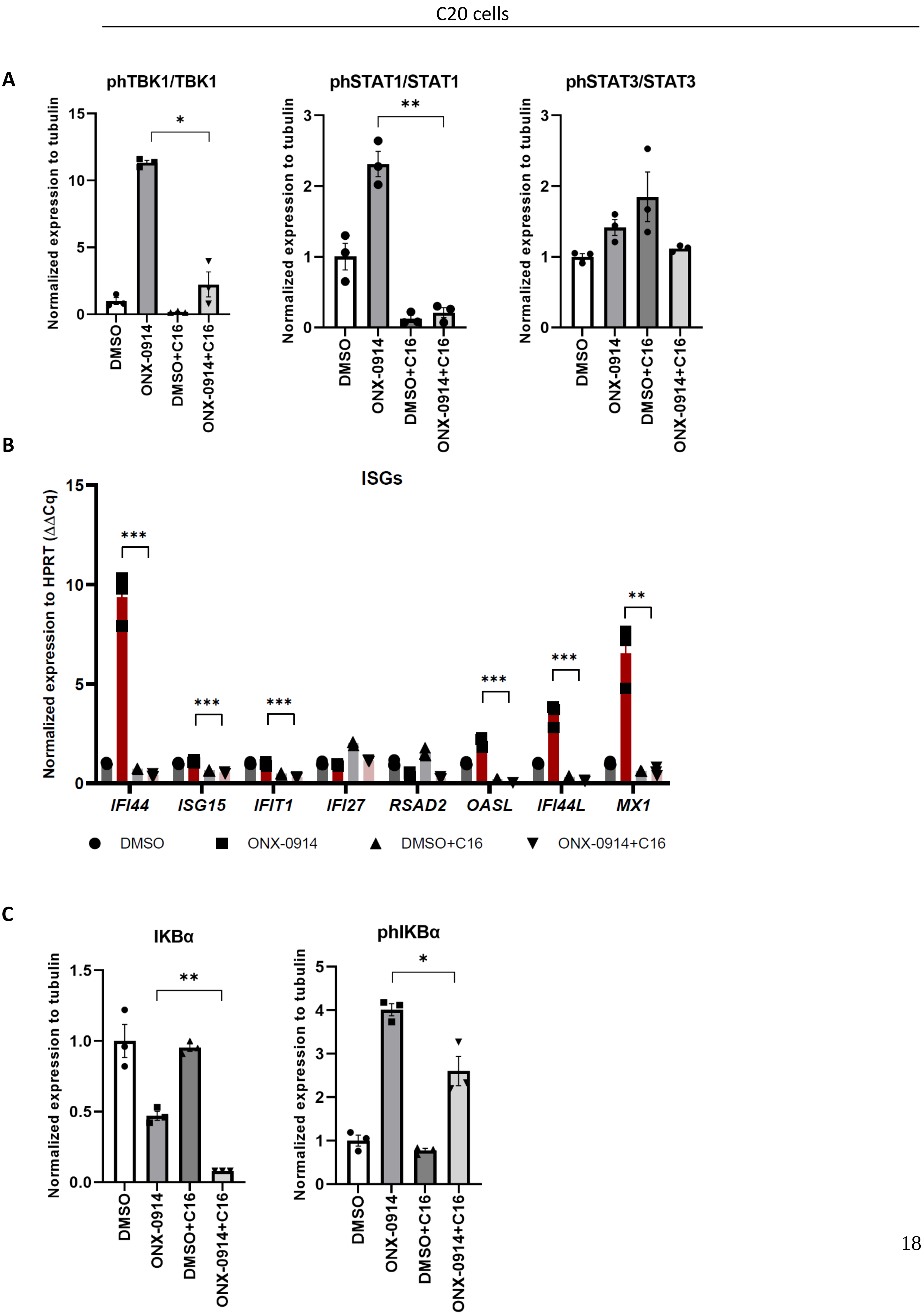

Supplementary Figure 12

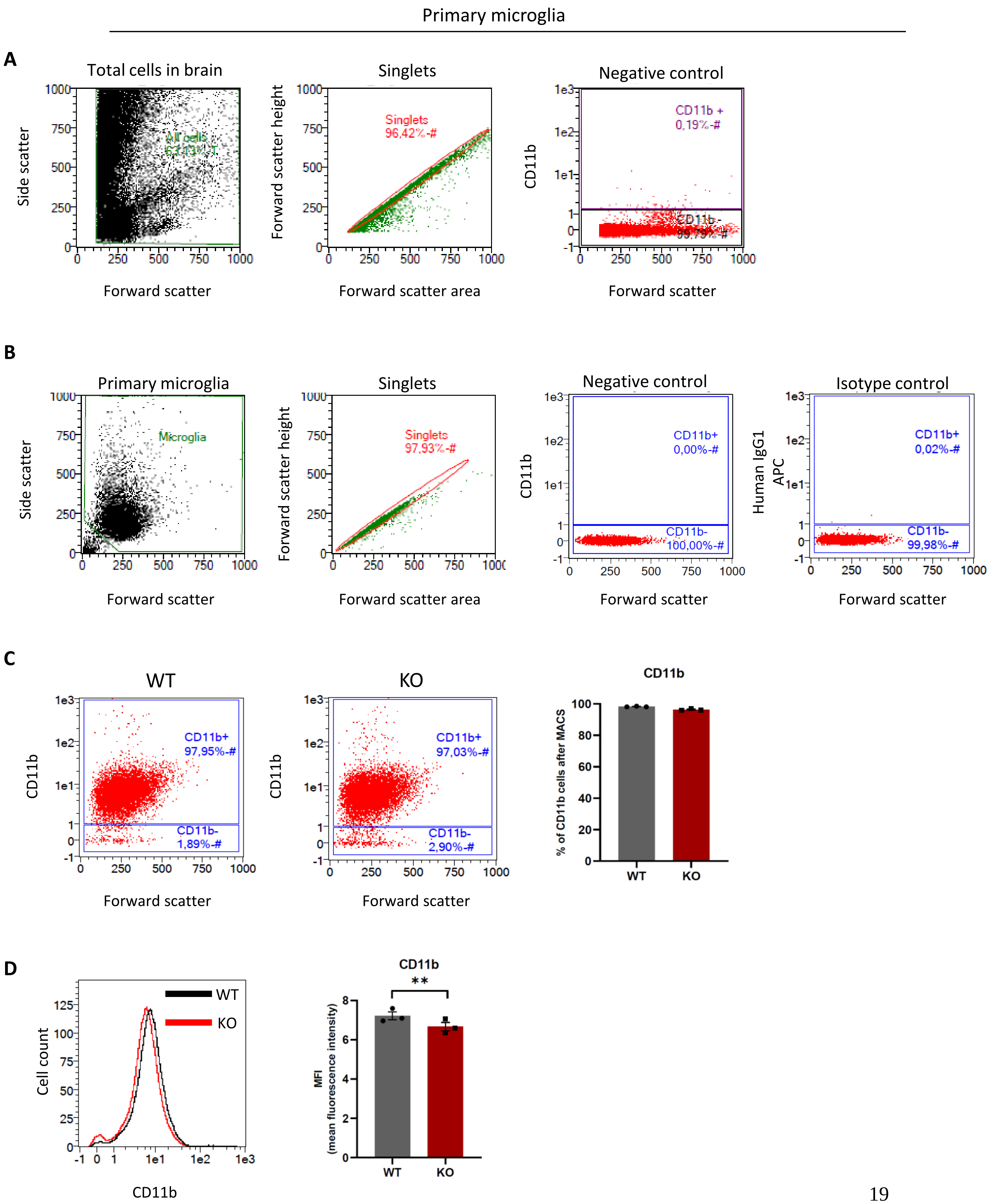

Supplementary Figure 13

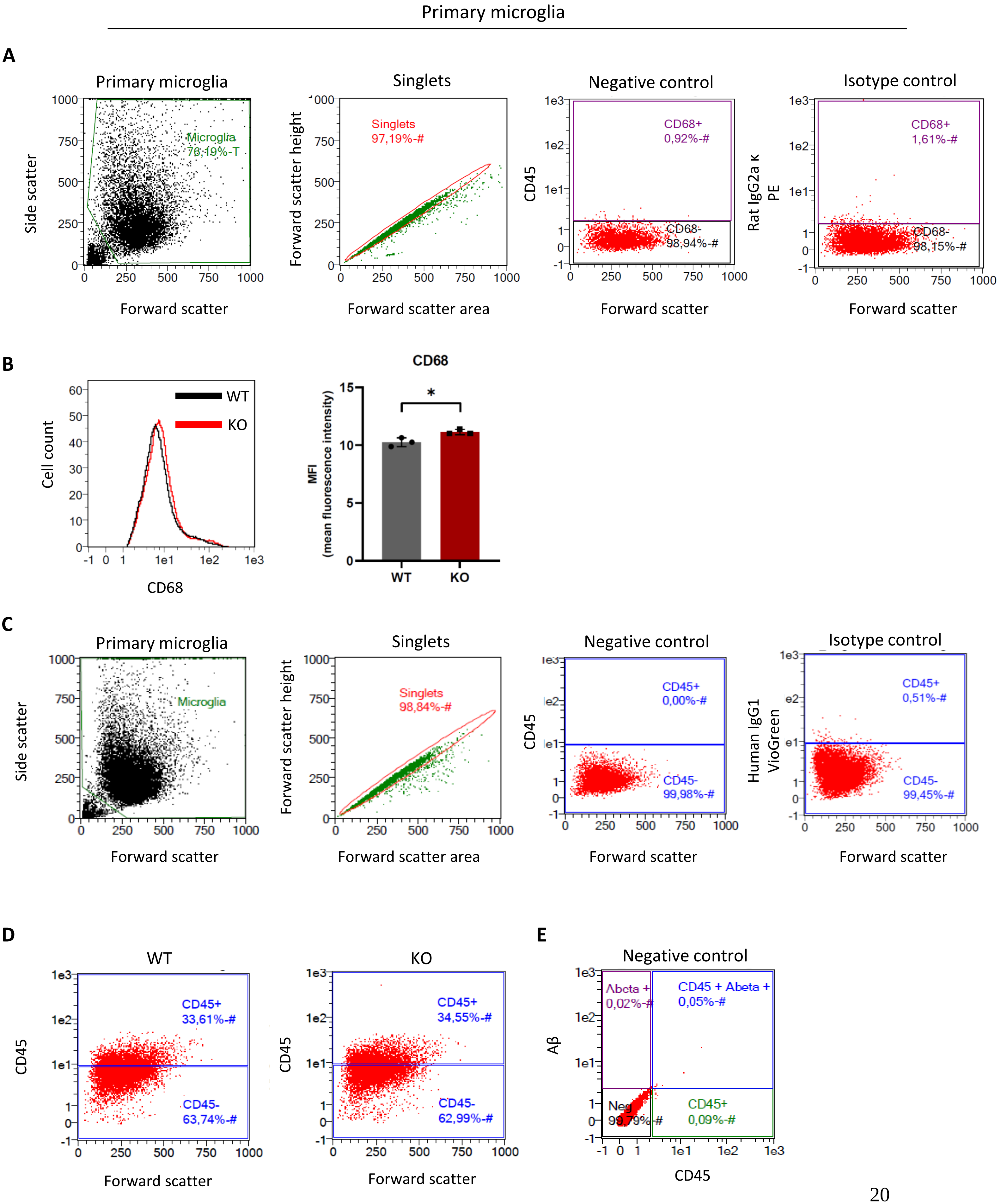

Supplementary Figure 14

A

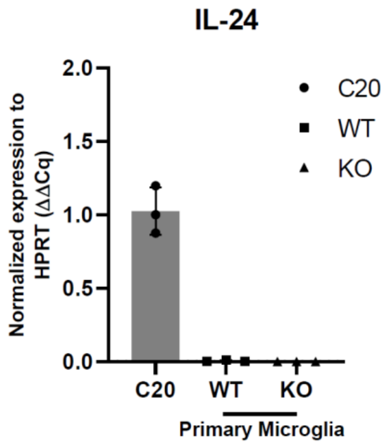

Supplement: Supplementary file 1 [file DataSheet_1.pdf]
